# Combined inhibition of JAK1/2 and DNMT1 by newly identified small-molecule compounds synergistically suppresses the survival and proliferation of cervical cancer cells

**DOI:** 10.1038/s41419-020-02934-8

**Published:** 2020-09-07

**Authors:** Shiqi She, Yang Zhao, Bo Kang, Cheng Chen, Xinyu Chen, Xiaobing Zhang, Wenjie Chen, Songsong Dan, Hangxiang Wang, Ying-Jie Wang, Jinhao Zhao

**Affiliations:** 1 State Key Laboratory for Diagnosis and Treatment of Infectious Diseases, Collaborative Innovation Center for Diagnosis and Treatment of Infectious Diseases, the First Affiliated Hospital, School of Medicine, Zhejiang University, 310003 Hangzhou, China; 2 Institute of Pesticide and Environmental Toxicology, Ministry of Agriculture Key Lab of Molecular Biology of Crop Pathogens and Insects, Zhejiang University, 310029 Hangzhou, China; 3 Key Laboratory of Combined Multi-Organ Transplantation, Ministry of Public Health, the First Affiliated Hospital, School of Medicine, Zhejiang University, 310003 Hangzhou, China

**Keywords:** Target identification, Translational research

## Abstract

Despite substantial advances in treating cervical cancer (CC) with surgery, radiation and chemotherapy, patients with advanced CC still have poor prognosis and significantly variable clinical outcomes due to tumor recurrence and metastasis. Therefore, to develop more efficacious and specific treatments for CC remains an unmet clinical need. In this study, by virtual screening the SPECS database, we identified multiple novel JAK inhibitor candidates and validated their antitumor drug efficacies that were particularly high against CC cell lines. AH057, the best JAK inhibitor identified, effectively blocked the JAK/STAT pathways by directly inhibiting JAK1/2 kinase activities, and led to compromised cell proliferation and invasion, increased apoptosis, arrested cell cycles, and impaired tumor progression in vitro and in vivo. Next, by screening the Selleck chemical library, we identified SGI-1027, a DNMT1 inhibitor, as the compound that displayed the highest synergy with AH057. By acting on a same set of downstream effector molecules that are dually controlled by JAK1/2 and DNMT1, the combination of AH057 with SGI-1027 potently and synergistically impaired CC cell propagation via dramatically increasing apoptotic cell death and cell-cycle arrest. These findings establish a preclinical proof of concept for combating CC by dual targeting of JAK1/2 and DNMT1, and provide support for launching a clinical trial to evaluate the efficacy and safety of this drug combination in patients with CC and other malignant tumors.

## Introduction

Cancer is a disease caused by abnormal proliferation and differentiation of cells that are governed by tumorigenic factors. It is the second most common cause of human death worldwide. Cervical cancer (CC) is the third most common gynecologic cancer, accounting for significant morbidity and mortality in women worldwide^1^. More than 85% of CC incidences and mortalities occur in developing countries, such as China, where diagnostic programs are still not well established^2^. Despite substantial advances in treating CC with surgery, radiation and chemotherapy, patients with advanced CC still have poor prognosis and significantly variable clinical outcomes due to tumor recurrence and metastasis^3^. Therefore, to develop more efficacious and specific treatments for CC is an unmet clinical need. Compared to the severe side effects and rapidly-acquired drug resistance issues associated with routine chemotherapies^4^, targeted therapies offer unique advantages including fewer side effects, improved drug effectiveness and hence improved quality of life. Thus far, only a few monoclonal antibody-based targeted therapies (e.g., Bevacizumab) are in clinical use for CC treatment, and a handful of small-molecule-based targeted therapies are at different phases of clinical trials^5^.

The JAK/STAT pathway that is highly activated in CC^6,7^ has emerged as an attractive target for cancer treatment. The signal transducer and activator of transcription (STAT) proteins are members of a family of transcription factors that mediate signals from cytokines and growth factors to regulate cell proliferation and differentiation^8^. Although STAT3 seems to act mainly as a transcription activator, transcriptional repression by STAT3 has also been described^9,10^. STAT activation is usually mediated by non-receptor tyrosine kinase members of the JAK family^11^. JAK/STAT signaling is continuously activated in various human carcinomas and promotes tumorigenesis and metastasis by upregulating the expression of genes encoding antiapoptotic proteins, cell-cycle regulators, and angiogenic factors^12–14^. It was also demonstrated that declined STAT3 induces apoptosis and G1/S and G2/M cell-cycle arrest in esophageal carcinoma by downregulating apoptosis and cell-cycle-related genes^15,16^. Moreover, inhibiting STAT3 activation led to reduced tumor growth and metastasis in different ovarian tumor models^17,18^. Therefore, targeting members of the JAK/STAT pathway is considered and evaluated as a promising therapeutic strategy for various human cancers. For instance, Ruxolitinib, an oral JAK 1/2 inhibitor, was newly demonstrated as a revolutionary treatment for patients suffering from intermediate/high-risk myelofibrosis^19^. In contrast, although the JAK/STAT activities can be effectively suppressed by a JAK inhibitor (JAKi) at doses lower than 100 nM, the inhibitory effect of JAKi on ovarian cancer cell propagation was relatively weak^17,20^. Likewise, newly identified JAK2-specific inhibitors^21,22^ also suffered relatively low drug efficacy for CC treatment. Thus, developing novel JAKis that can exhibit higher efficacy against CC and overcome drug resistance to existing JAKis is a worthy endeavor.

Traditional drug development is a lengthy, time consuming, and costly process with a very low success rate. With the continuous advancement of computing technology, computer-aided drug design has become a popular method for preclinical drug research with its unique advantages of high efficiency and low cost^23^. Novel core structure LibDock Model^24^, CHARMM force field^25^, and CDOCKER molecular docking^26^ were reported as an effective virtual screening strategy. In this study, we employed a combinatorial strategy of high-throughput virtual screening and kinase activity prediction, and identified AH057 as a novel JAK1/2 inhibitor that blocks interleukin 6 (IL-6)-, interferon-α (IFN-α)-, and interferon-γ (IFN-γ)-induced JAK1, JAK2, and protein-tyrosine kinase 2 (TYK2) phosphorylation and the corresponding STAT1 and STAT3 phosphorylation. Accordingly, AH057 exhibited potent antitumor activities including compromised cell proliferation and invasion, increased apoptosis and cell-cycle arrest, and impaired tumor progression in the xenograft model. Furthermore, we screened a chemical library comprising 2094 small molecules to identify compounds work synergistically with AH057 in halting CC cell propagation. We discovered that the combination of AH057 and a DNMT1 inhibitor SGI-1027 displayed strong synergistic antitumor effect against CC cells, raising the possibility that synergistically targeting JAK/STAT and DNMT1 might be a promising strategy for effectively treating CC and other human cancers.

## Results

### Virtual screening of small-molecule compounds targeting JAK2

**Fig. 1:**
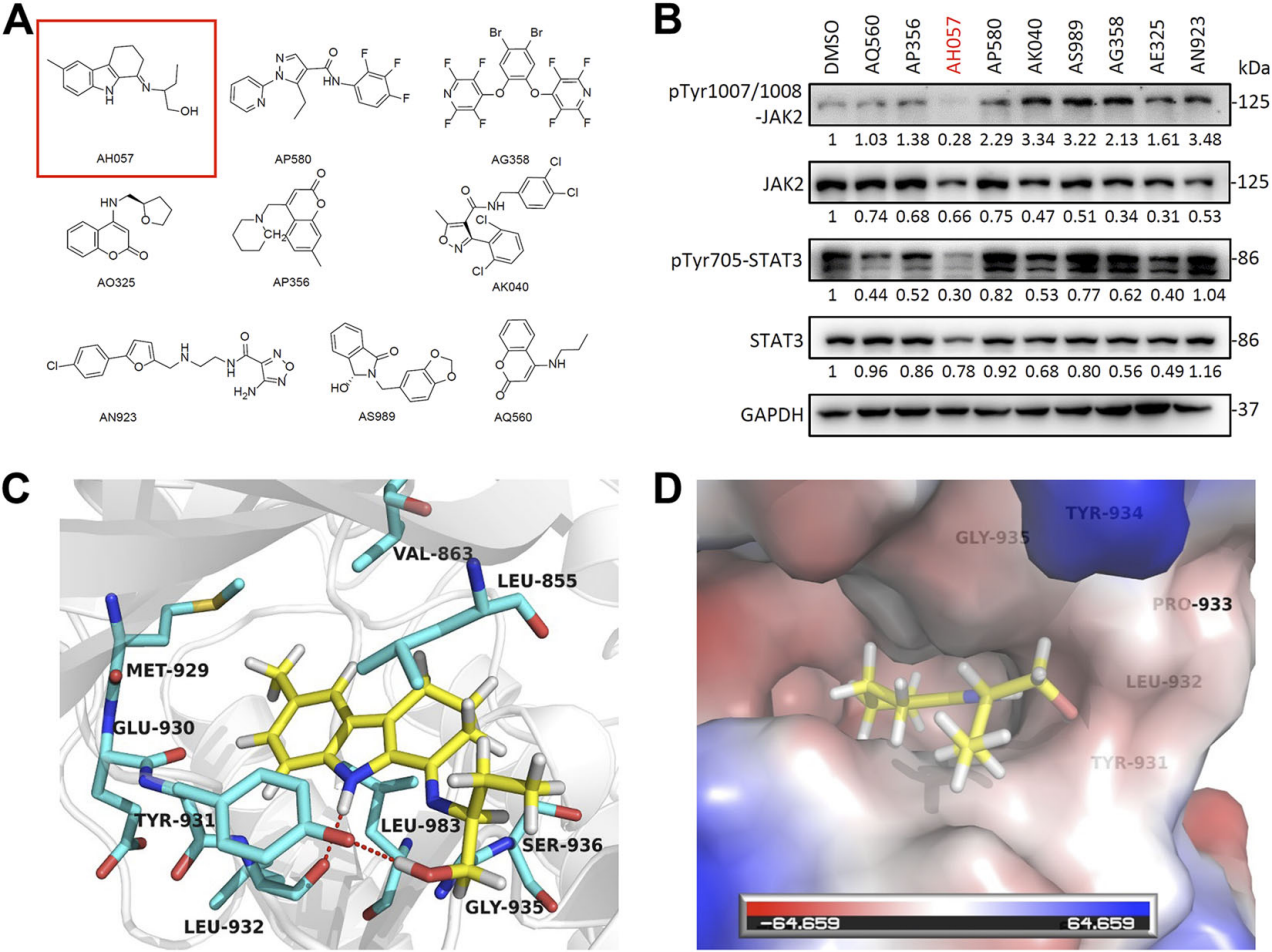
Docking-based virtual screening of JAK inhibitors. **a** The chemical structures of nine top-ranking compounds. **b** Biochemical validation of top-ranking compounds. HeLa cells were treated with nine top-ranking compounds for 4 h and western blotted with indicated antibodies. All compounds were added to the final concentration of 10 μM. The data were from one of three independent experiments that gave similar results, the band intensities were quantified by densitometry. **c** Docking model of compound AH057 binding to JAK2 (PDB code: 3RVG). Key JAK2 interacting residues are shown in cyan sticks. Compound AH057 is shown as stick, with carbon atoms colored in yellow, nitrogen atoms in blue, and oxygen atoms in red. Hydrogen bonds were shown in red dashed lines. **d** Solvent-accessible surfaces of the binding pocket of JAK2 for AH057.

In this experiment, 20,000 potential small-molecule compounds, from the SPECS small-molecule database, were assessed by high-throughput virtual screening for their likelihood of direct binding to JAK2. Based on the docking scores and binding energies, the top 20 ranking small-molecule compounds were selected. Then, taking into account the diversity of their chemical structures, we selected nine compounds with the highest scores (Fig. 1a) out of which AH057 ranked at the top. Furthermore, western blot (WB) analysis verified that among the nine candidate compounds, AH057 exhibited the highest activity in reducing JAK2 phosphorylation and the phosphorylation of STAT3, an immediate downstream target of JAK2 (Fig. 1b).

### Molecular docking elucidated the binding mode of AH057 with JAK2

In order to further elucidate the binding mode of AH057 with JAK2, molecular docking was carried out by using Accelrys Discovery Studio (version 2.5, San Diego, USA). Key residues of the active site pocket were highlighted. The lowest-energy binding conformation of AH057 with JAK2 was shown (Fig. 1c). AH057 was docked into the active site pocket of JAK2 in a manner similar to its ligand molecules. The pocket is mainly composed of crucial amino acid residues Leu855, Val863, Glu930, Tyr931, Leu932, and Leu983. The benzene ring in the AH057 molecule is inserted into the hydrophobic pocket and has a π–π interaction with the Tyr931 residue benzene ring. At the same time, amino hydrogen and branched chain hydroxyl hydrogen form strong hydrogen bonds with Tyr931 and Leu932 residues (Fig. 1d), indicating that AH057 can bind well to the active site of JAK2.

### AH057 potently blocked STAT3 phosphorylation by directly inhibiting JAK2

**Fig. 2:**
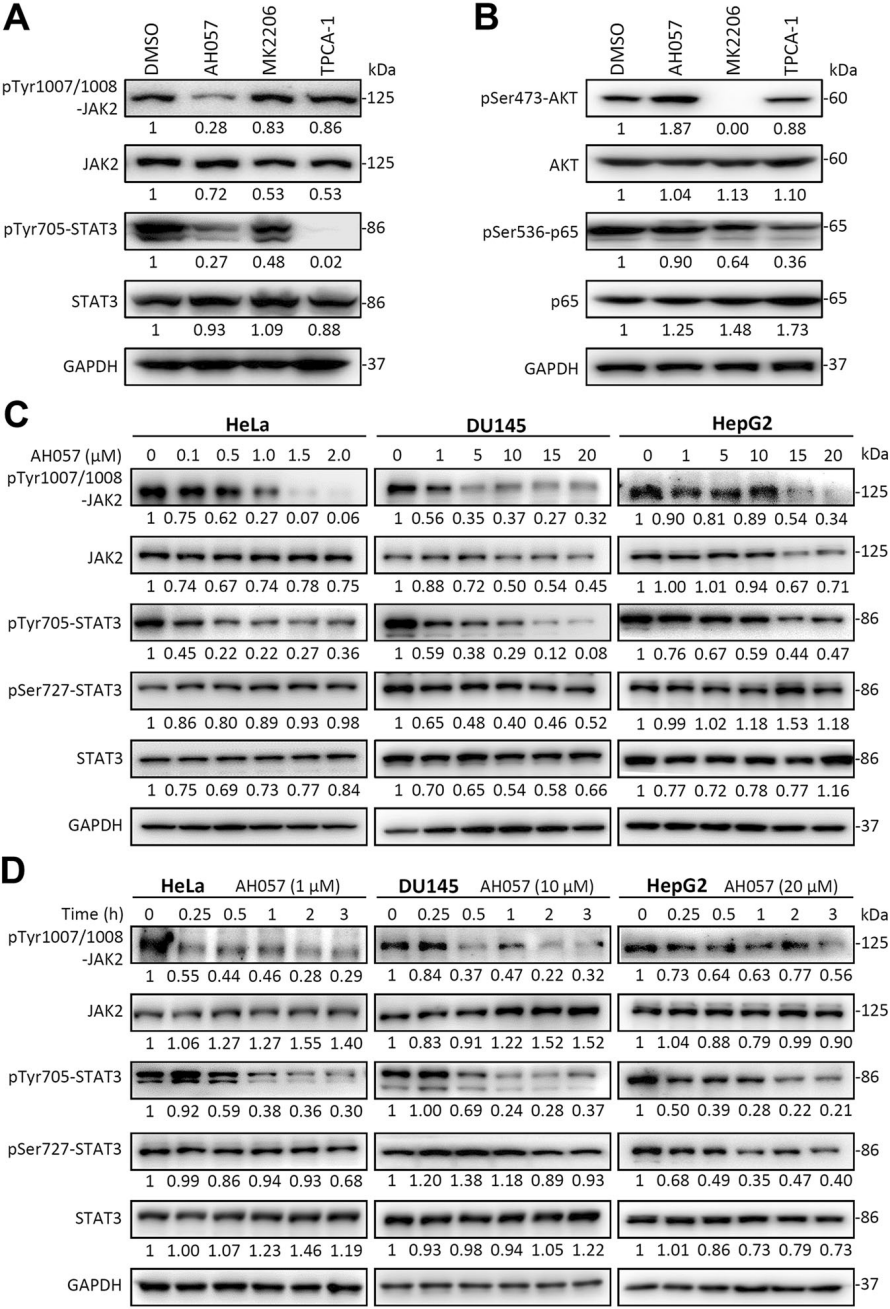
AH057 inhibited JAK2/STAT3 in multiple cancer cell lines. **a** Effects of AH057 and two known AKT and IKK-2 inhibitors on JAK2/STAT3 in HeLa cells were assessed by WB. **b** Effects of AH057 and two known AKT and IKK-2 inhibitors on AKT and p65 in HeLa cells were assessed by WB. The concentration of all inhibitors in (**a**, **b**) was 1 μM. **c** HeLa, DU145, and HepG2 cells were treated with indicated concentrations of AH057 for 2 h, and cell lysates were western blotted by indicated antibodies. **d** HeLa, DU145, and HepG2 cells were treated with AH057 for indicated time points, and cell extracts were analyzed by WB. DMSO was used as a control. The data in (**a–d**) were from one of three independent experiments that gave similar results. All band intensities were quantified by densitometry.

It has been well established that IL-6/JAK is a crucial node regulating the STAT/PI3K/NF-κB signaling pathways of several essential biological processes^27^. To evaluate and compare the effect of AH057 on STAT3, PI3K and NF-κB signaling pathways, AH057, an AKT inhibitor MK2206 or an NF-κB inhibitor TPCA-1 was individually applied to HeLa cells. The results of WB analysis showed that AH057 significantly inhibited the phosphorylation of JAK2-Tyr1007/1008 and STAT3-Tyr705 in HeLa cells (Fig. 2a), but not that of AKT-Ser473 or p65-Ser536 (Fig. 2b), indicating that AH057 selectively inhibited the JAK2–STAT3 signaling without affecting the PI3K–AKT pathway and NF-κB pathway in CC cells.

To further confirm the inhibitory effect of AH057 on STAT3 phosphorylation, HeLa, DU145, and HepG2 cells, which contain continuously activated STAT3 and the IL-6 autocrine loop, were selected for the following experiments. Cells were treated with the indicated concentrations of AH057 for 2 h, and the WB results revealed that JAK2 and STAT3 activities were remarkably inhibited in all three cell lines. In these cell lines, the levels of phosphorylated STAT3-Tyr705 and JAK2-Tyr1007/1008 were attenuated in a dose-dependent manner (Fig. 2c), verifying the specific inhibitory effect of AH057 on JAK2–STAT3 signaling. Among the three cell lines, CC cell line HeLa showed the highest drug sensitivity. We then determined the kinetic aspect of AH057 and found that it suppressed JAK2 and STAT3 activation in a time-dependent manner (Fig. 2d). Importantly, AH057 rapidly inhibited JAK2 phosphorylation in as short as 15 min, fully supporting the scenario in which AH057 directly binds JAK2 and inhibits its enzymatic activity and downstream signaling cascades.

### AH057 broadly inhibited JAK/STAT signaling pathways activated by multiple sources

**Fig. 3:**
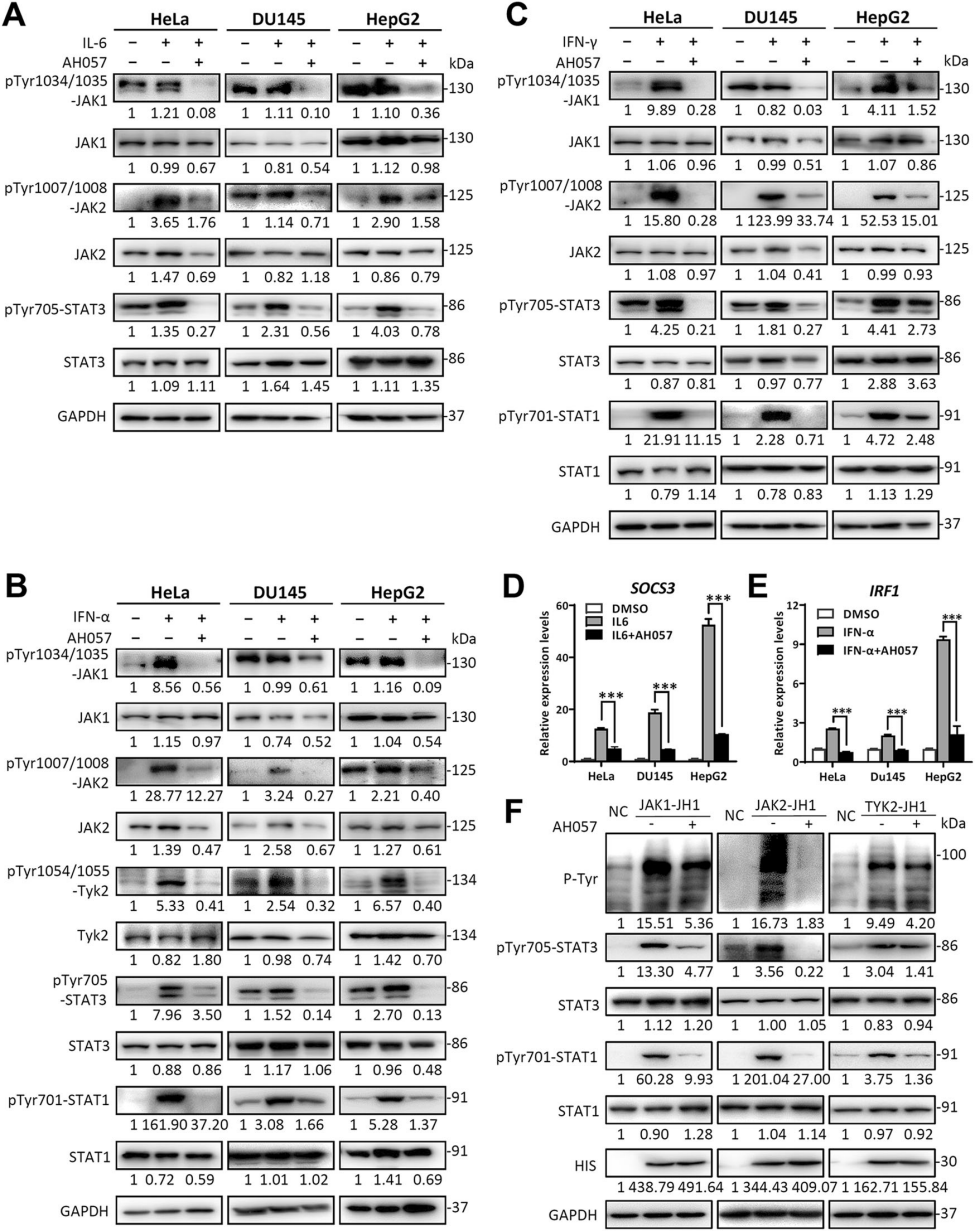
AH057 inhibited JAK1/JAK2 and their downstream targets in multiple cancer cell lines. HeLa, HepG2, and DU145 cells were cultured in MEM medium with 0.2% FBS overnight. Serum-starved cells were pretreated with DMSO or various concentrations of AH057 (as indicated in Fig. 2d) for 1 h, further stimulated with (**a**) IL-6 (250 ng/ml), (**b**) IFN-α (250 ng/ml), or (**c**) IFNγ (250 ng/ml) for 1 h, and cell lysates were blotted by indicated antibodies. **d–f** HeLa, DU145, and HepG2 cells were serum starved and pretreated with AH057 for 1 h and stimulated by IL-6 (250 ng/ml) or IFNα (250 ng/ml) for 4 h. Relative mRNA levels of SOCS3 (**d**) and IRF1 (**e**) were analyzed by qRT-PCR. **f** AH057 blocked the phosphorylation of the JAKs-JH1 tyrosine kinase domains overexpressed in HEK293T cells and their downstream STAT1/STAT3. Cells were treated with AH057 (20 μM) for 4 h, and cell lysates were blotted with indicated antibodies. The data in (**a–c**) and (**f**) were from one of two to three independent experiments that gave similar results. Results shown in (**d–e**) were representative of three independent experiments. Two-tailed unpaired Student’s *t* tests were used for statistical analyses. *P < 0.05; **P < 0.01; ***P < 0.001 were vs. Control (the DMSO group). All band intensities were quantified by densitometry.

To assess and compare the effects of AH057 on JAK/STAT signaling pathways elicited by different upstream stimuli, we treated HeLa, DU145 and HepG2 cells with either IL-6 or interferons (IFNs) that activate STATs via IL-6 receptor/gp130 or type I/II interferon receptors, respectively. Firstly, since IL-6 can activate STAT3 through JAK1 and/or JAK2, we measured the phosphorylation state of both JAKs. Clearly, AH057 effectively blocked IL-6-induced phosphorylation of both JAK1 and JAK2 and their downstream STAT3 phosphorylation (Fig. 3a). Secondly, IFNα is known to activate STAT1 and STAT3 through JAK1, JAK2, and TYK2, and we found that IFNα-stimulated phosphorylation of JAK1, JAK2, TYK2, STAT1, and STAT3 were all decreased by AH057 treatment (Fig. 3b). Thirdly, IFNγ can activate STAT1 and STAT3 through JAK1 and/or JAK2, and our results indicated that AH057 not only blocked IFNγ-induced phosphorylation of STAT1 and STAT3 but also directly suppressed IFNγ-activated phosphorylation of JAK1 and JAK2 (Fig. 3c). All of these results were obtained in 1 h after administration of cytokine and IFN, indicating that AH057 directly blocks JAK1, JAK2, and TYK2 activation. Considering JAK/STAT signaling controls cell survival and proliferation by regulating their downstream genes, it is important to evaluate the effect of AH057 on the expression of the JAK/STAT target genes. Taking as an example, the mRNA level of suppressor of cytokine signaling 3 (SOCS3), a direct target gene of STAT3, was examined by qRT-PCR. The result revealed that AH057 dramatically diminished SOCS3 mRNA transcription induced by IL-6 in all three cell lines (Fig. 3d). Likewise, IFNα-induced transcription of the two STAT1 target genes, interferon regulatory factor 1 (IRF1) and interferon regulatory factor 2 (IRF2), was also suppressed by AH057 in all three cell lines (Figs. 3e, S1E). In order to determine whether AH057 directly inhibits JAK kinase activity biochemically, we individually overexpressed the JH1 domains of JAK1, JAK2, or TYK2 in HEK293T cells. In these cells, total tyrosine phosphorylation levels were remarkably elevated. Exposure of JAKs-JH1 overexpressing cells to AH057 resulted in a significant decrease in total tyrosine phosphorylation. Similarly, overexpressing the JH1 domain of JAK1, JAK2, or TYK2 led to increased phosphorylation of STAT1 and STAT3, which was all inhibited by AH057 (Fig. 3f). To summarize, AH057 is a novel and direct JAK1/2 inhibitor that can potently block the JAK/STAT signaling pathways induced by multiple extracellular factors.

### AH057-mediated JAK/STAT inhibition was associated with attenuated proliferation and migration of somatic cancer cells

**Fig. 4:**
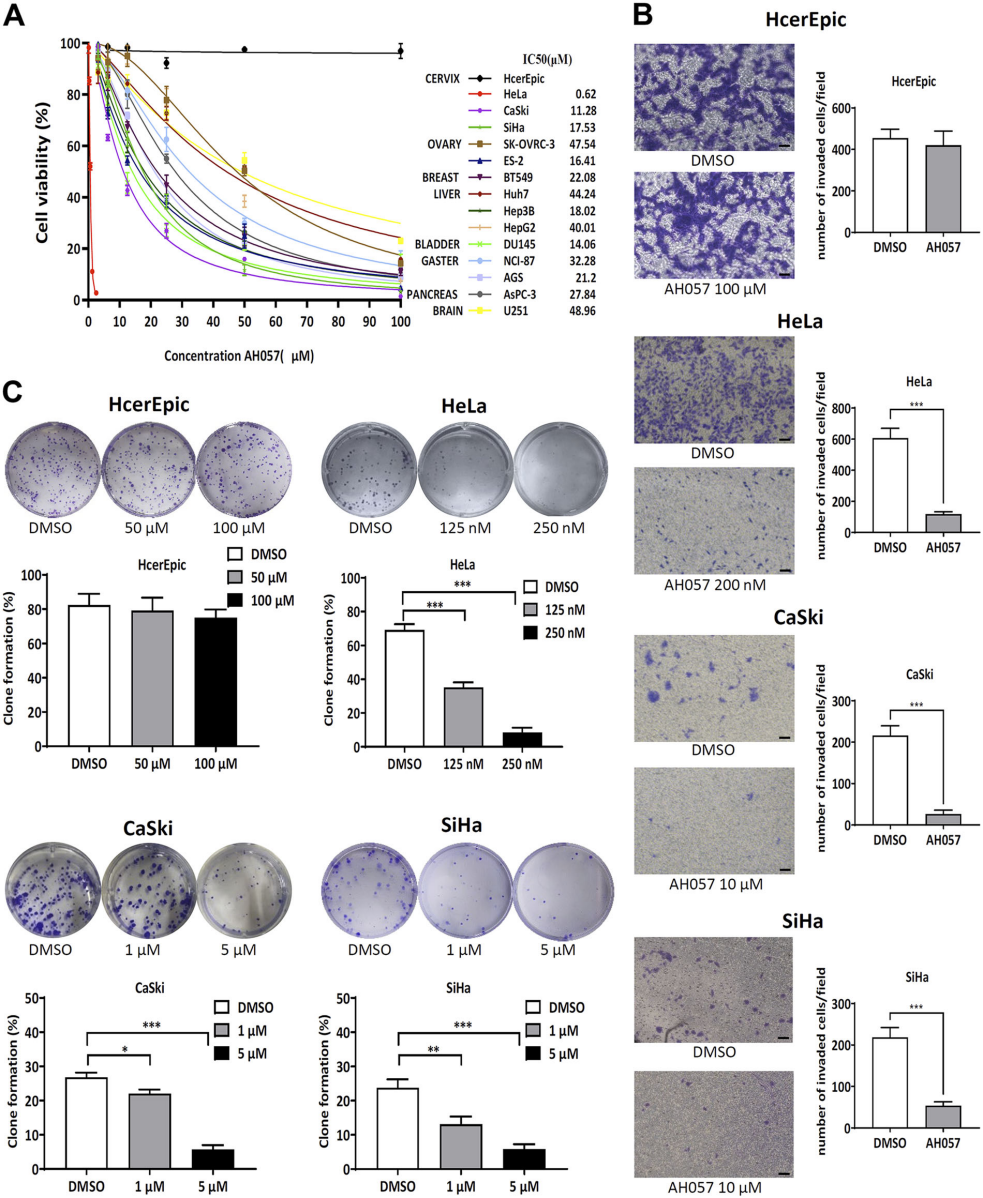
AH057-mediated JAK/STAT inhibition was associated with growth retardation and migration defects of somatic cancer cells. **a** Cell viability and IC50 values for indicated cell lines treated with AH057 or DMSO for 72 h were determined by CCK-8 assay (*n* = 3). **b** Effect of AH057 on CC cell invasion determined by transwell assay. Scale bars, 100 µm. **c** Effect of AH057 on CC cell proliferation determined by colony formation assay. Results shown in (**b**, **c**) were representative of three independent experiments. Two-tailed unpaired Student’s *t* tests were used for statistical analyses. *P < 0.05; **P < 0.01; ***P < 0.001 were vs. Control (the DMSO group).

JAK/STAT signaling regulates survival, proliferation, migration as well as invasion of cancer cells, which has been confirmed by numerous studies. In this study, we first evaluated the proliferation of a panel of human cancer cell lines to characterize the spectrum of activity of AH057. Among all the tested cell lines, the human cervical cell lines HeLa and CaSki were the most sensitive in terms of IC50 values (Fig. 4a). Next, to investigate whether AH057 may dampen the invasion ability of CC cells, an in vitro invasion test was performed. HcerEpic, HeLa, CaSki, and SiHa cells were treated with relatively low concentrations of AH057 and representative photomicrographs of cells (×100) were taken and analyzed. AH057 impaired the invasion ability of CC cells but not normal HcerEpic cells (Fig. 4b). Furthermore, we performed colony formation assay to evaluate the long-term impact of AH057 on cell proliferation. As shown in Fig. 4c, AH057 treatment significantly decreased the numbers of CC cell colonies in a dose-dependent manner. Finally, AH057 significantly inhibited cell motility as reflected in the scratch assay (Fig. S3). Taken together, AH057 led to significantly attenuated cell proliferation and migration by inhibiting the JAK/STAT pathway.

### AH057-induced apoptotic death and cell-cycle arrest of somatic cancer cells

Since AH057 significantly compromised cell viabilities of a variety of cancer cells (Fig. 4a), we hypothesized that the decrease in cell viabilities might be due to apoptotic cell death. Flow cytometry verified that AH057 treatment markedly increased apoptosis rates (Fig. S2A, B). Furthermore, we conducted cell-cycle analysis of AH057-treated HeLa and CaSki cells by flow cytometry and the data were shown in Fig. S2C, D. The cells subjected to increasing concentrations of AH057 were arrested at G1 phase with increasing percentages. For instance, the percentage of cells at G1 phase was 69.95%, 77.61%, and 81.9% for control, 500 nM AH057-treated, and 1 μM AH057-treated HeLa cells, respectively (Fig. S2D).

### AH057-induced JAK1/2 inhibition was associated with growth retardation and apoptosis of somatic cancer cells in vivo

**Fig. 5:**
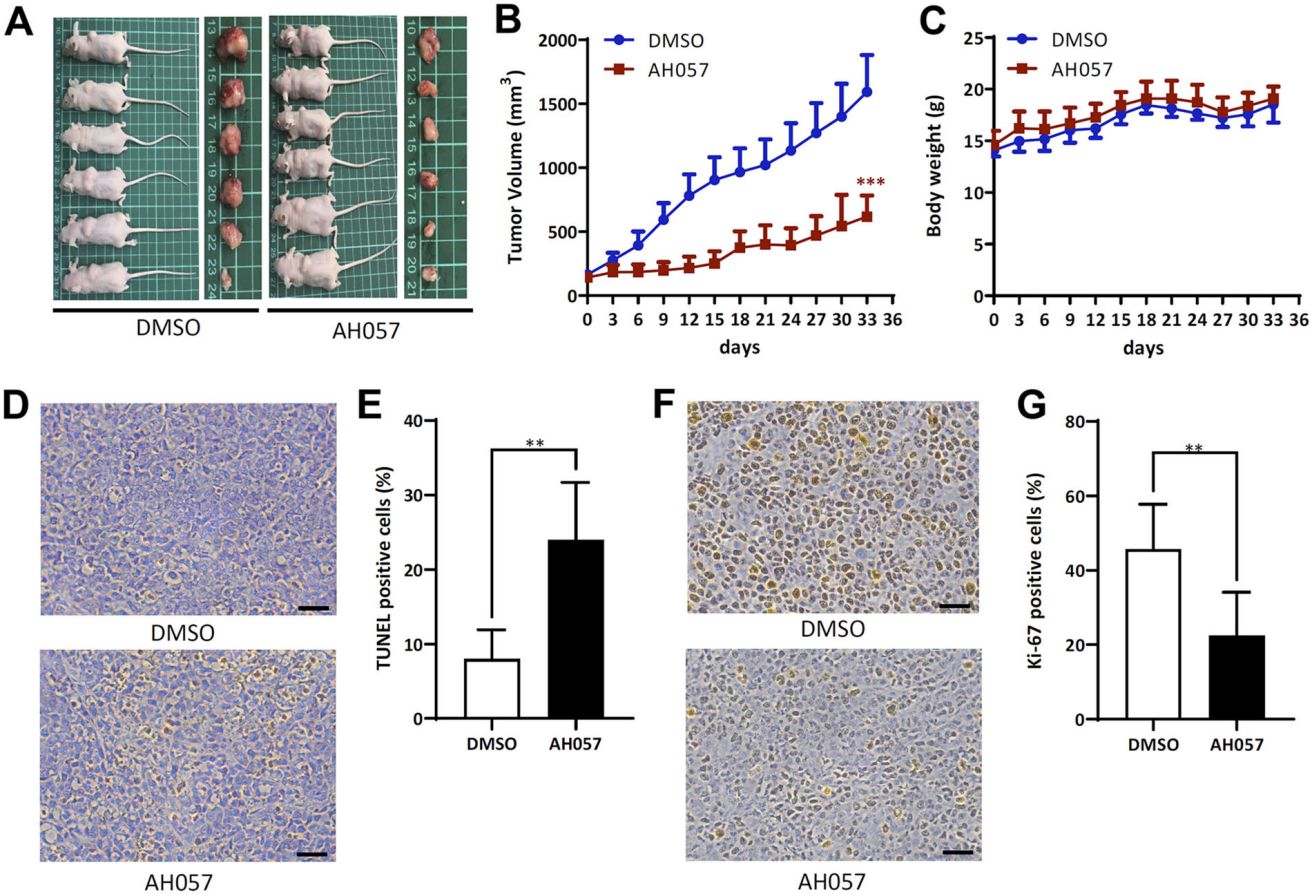
Orally-administered AH057 inhibited propagation of HeLa cell xenografts in vivo. **a** The BALB/c nude mice were orally administered with AH057 or vehicle as detailed in the “Materials and methods”. At the end of the experiments, the executed mice bearing the tumors (left) and the excised tumors (right) were photographed and presented. **b**, **c** Effect of AH057 on tumor volume and body weight changes in xenograft mice. **d** Representative images of tumor samples from vehicle (DMSO)- and AH057-treated mice analyzed by the TUNEL assay. Scale bars, 50 µm. Nuclei of apoptotic cells were stained in brown, whereas normal nuclei remained blue. **e** Quantification of the TUNEL assay results for vehicle- and AH057-treated tumor samples. **f** Immunohistochemical staining of Ki-67 in tumor samples from vehicle- and AH057-treated mice. Scale bars, 100 µm. **g** Quantification of the Ki67 staining results for vehicle- and AH057-treated tumor samples. Results shown in (**b**, **c**, **e**, **g**) were representative of 6 independent experiments. 2-way ANOVA tests were used for statistical analyses. *P < 0.05; **P < 0.01; ***P < 0.001 were vs. Control (the DMSO group).

We further evaluated the in vivo antitumor efficacy of AH057 on HeLa cells in a BALB/c nude mouse xenograft model. When the xenografted tumors reached relatively small volumes (~100 mm^3^), the vehicle (DMSO) or AH057 was administered intragastrically (50 mg/kg/day, respectively) for 30 consecutive days, immediately followed by tumor excision and analyses (Fig. 5a). Clearly, AH057 treatment significantly reduced tumor volumes (Fig. 5b). Besides, AH057 was well tolerated in all groups with no mortality or significant loss of body weight observed during the trial (Fig. 5c). TUNEL staining of the tumor tissues indicated that AH057-induced apoptosis of xenografted tumor cells (Fig. 5d, e). The immunohistochemistry showed that the number of cells positive for Ki67 staining in AH057 group was markedly lower than that in the control group (Fig. 5f, g), indicating the inhibition of cell proliferation.

### Drug combination screening for AH057 and synergistic inhibitory effects of AH057 and SGI-1027 on HeLa cells

**Fig. 6:**
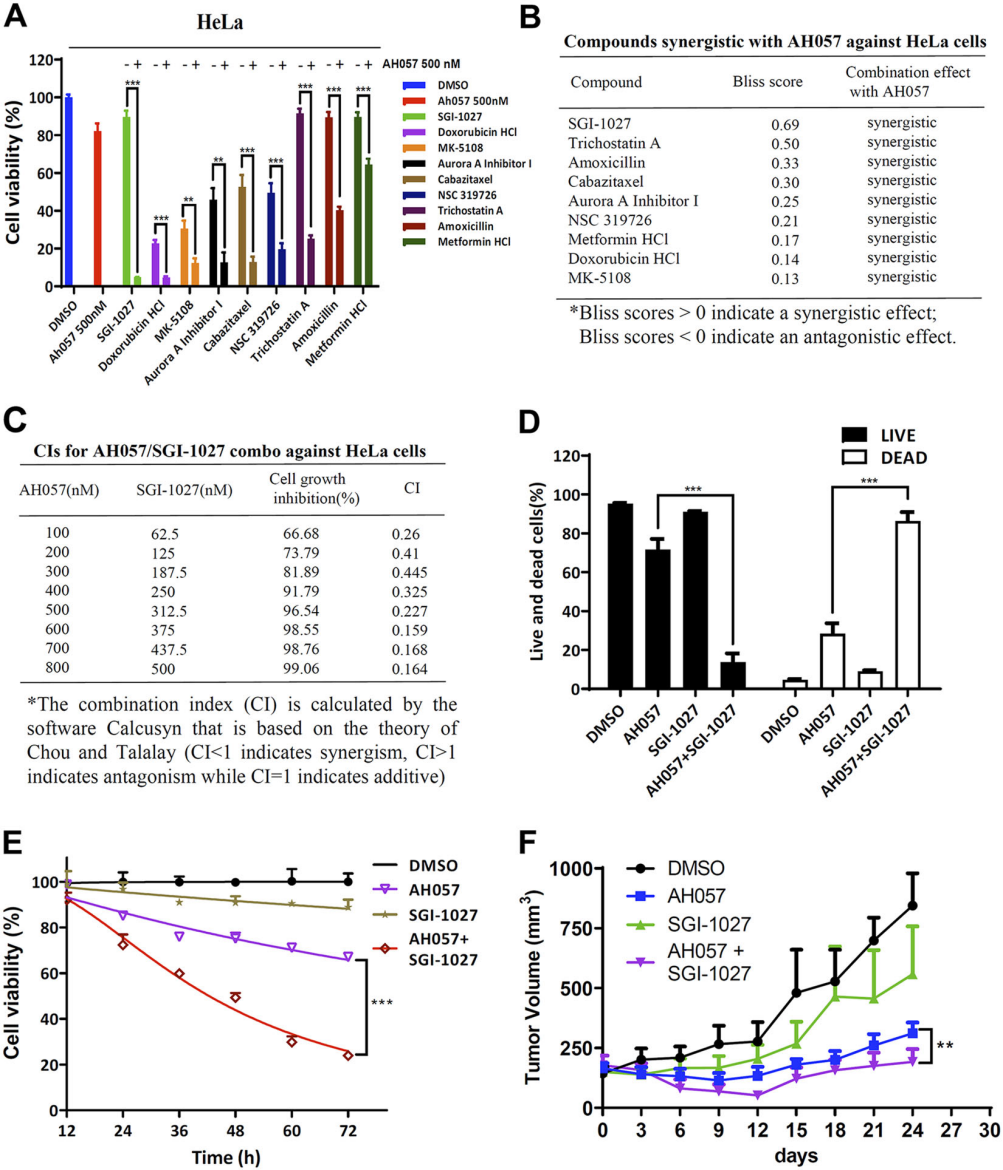
Identification of synergistic compounds for AH057 and the effects of combined treatment with AH057 and SGI-1027 on HeLa cell propagation. **a** The viability of HeLa cells treated with AH057 combined with candidate compounds (*n* = 3) was measured by CCK-8 assay, and the data for selected hits were presented. **b** Bliss analyses for the combination of AH057 with selected compounds in inhibiting the proliferation of HeLa cells. **c** CI analyses for the AH057/SGI-1027 combination at different doses against HeLa cells. **d** AH057 and SGI-1027 combo potently induced cell death of HeLa cells. See Fig. S4 for more details. **e** The combination effect of AH057 and SGI-1027 was time dependent. HeLa cells were treated with AH057 (500 nM) and/or SGI-1027(1 μM) for 48 h. **f** Effect of orally-administered AH057, SGI-1027, and their combo on tumor volume changes in xenograft mice. Accompanying data were shown as Fig. S10. Two-tailed unpaired Student’s *t* tests were used for statistical analyses. *P < 0.05; **P < 0.01; ***P < 0.001.

Despite we have successfully identified a novel JAK1/2 inhibitor, like all the other JAKis, AH057 only inhibited cancer cell proliferation at relatively high concentrations. This prompted us to search for molecular pathways and compounds that may act synergistically with AH057 in inhibiting the propagation of CC cells. To this end, AH057 was combined individually with compounds from a biologically annotated compound library provided by Selleck. The concentration of AH057 used for the screening was set at 500 nM where HeLa cell growth was partially inhibited. The concentration of library compounds applied was set at 1 μM where each compound was considered to have certain degree of inhibitory effect (Fig. 6a). According to the screening results, the tested library compounds were classified by Bliss independent model as synergistic, additive/independent or antagonistic to the effect of AH057. This model is widely used to compare the combined and individual effects of different drugs. Detailed definitions of three categories are described in the “Materials and methods”. Based on the Bliss expectation scores, top nine potential small-molecule compounds were identified of which SGI-1027 showed the best synergistic effect (Fig. 6b). In order to better characterize the combinatory effects, CI (combination index) analyses of the combination of AH057 and SGI-1027 with a concentration gradient in HeLa cells were performed (Figs. 6c, d, S4). At all eight concentrations, CIs of AH057 and SGI-1027 were well below 1 (Fig. 6c), indicating strong synergism. In addition, the time course result of the combined effect of AH057 and SGI-1027 was shown in Fig. 6e. The combined effect of the two compounds was also confirmed in a mouse xenograft model (Fig. 6f) where the cardiac and renal toxicity of the compounds were inappreciable (Fig. S10). Strong synergism was also seen when SGI-1027 was combined individually with other existing JAKis (Fig. S4). In addition, the synergistic effect of AH057 and SGI-1027 also held in other types of cancer cells in a dose-dependent manner (Figs. S5–S7).

### Combination of AH057 and SGI-1027 dramatically induced apoptotic death and cell-cycle arrest of HeLa cells

**Fig. 7:**
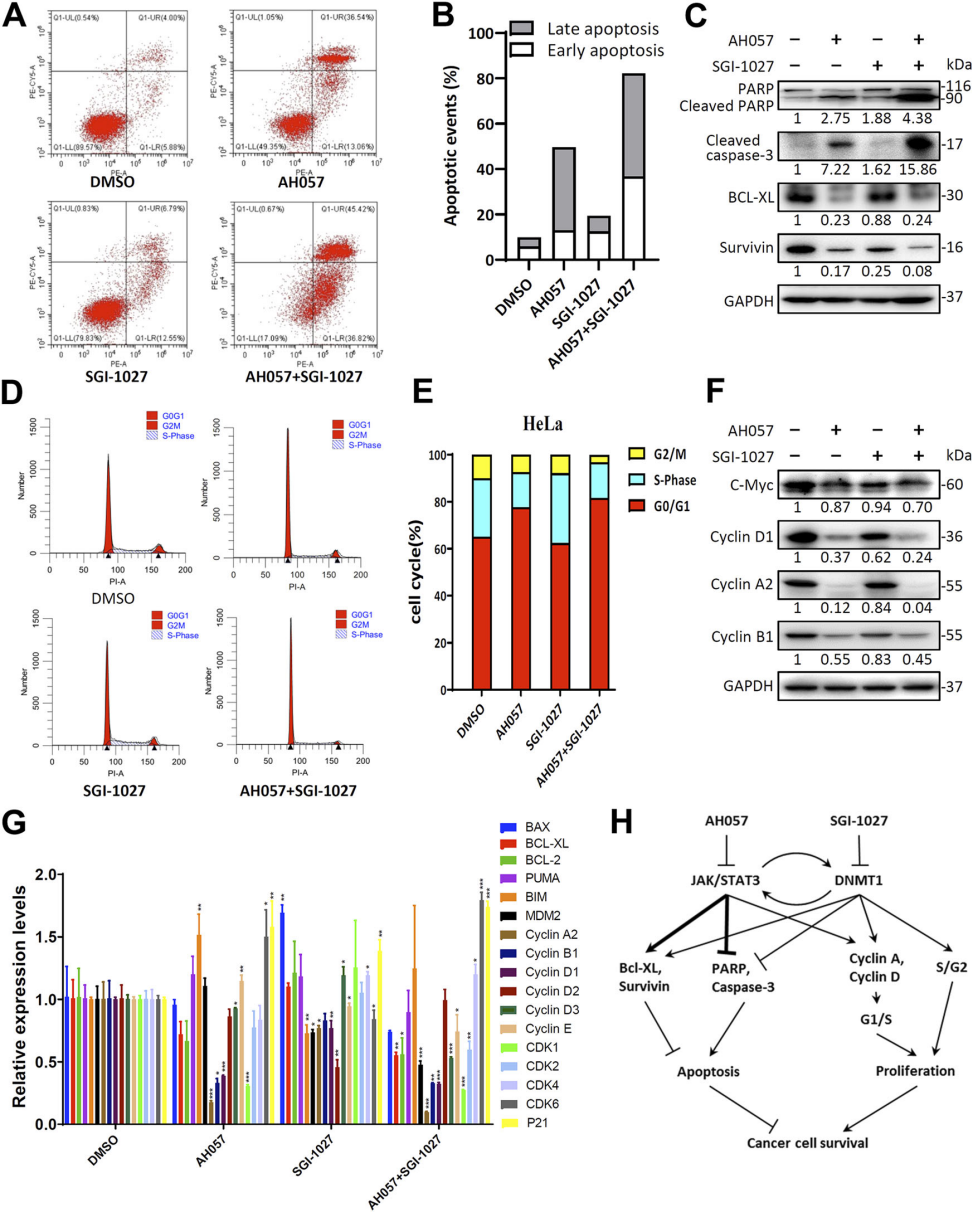
Combination treatment of AH057 and SGI-1027 synergistically induced apoptotic cell death and cell-cycle arrest of HeLa cells. **a** Flow cytometry analysis of apoptotic death of HeLa cells subjected to AH057 and SGI-1027 treatment. Cells were treated with either drug alone (500 nM of AH057, 1 μM of SGI-1027) or in combination for 24 h, and stained with annexin-PE(x-axis)/7-AAD(y-axis). **b** Cells in four groups that underwent early apoptotic cell death (bottom right quadrants) and late apoptotic cell death (top right quadrants) were calculated and plotted as percentages of total cells. **c** Key apoptosis-related proteins in the above-treated HeLa cells were analyzed by WB, the band intensities were quantified by densitometry. **d** The above-treated HeLa cells were fixed in 70% of ethanol, stained with propidium iodide, and subjected to flow cytometry for cell-cycle analysis. **e** Quantitation of the percentages of cells in different cell-cycle phases. **f** Key cell-cycle-related proteins in the above-treated HeLa cells were analyzed by WB, the band intensities were quantified by densitometry. Results shown in (**a–f**) were representative of three independent experiments. **g** Relative mRNA levels of key apoptosis- and cell-cycle-related genes in the above-treated HeLa cells. The data were expressed as mean ± S.D. of triplicate measurements from one of three independent experiments, which gave similar results. Two-tailed unpaired Student’s *t* tests were used for statistical analyses. *P < 0.05; **P < 0.01; ***P < 0.001. **h** Working model for synergistic effects of AH057 and SGI-1027 on somatic cancer cells. Lines with arrowheads indicate positive regulation, while bar-headed lines indicate negative regulation. Bold lines indicate main regulation routes.

To gain insights into the molecular mechanisms underlying the compromised cell viability, the mRNA levels of typical apoptosis and cell-cycle related genes in AH057 and SGI-1027 treated cells were determined by qRT-PCR, and the genes exhibited most significant changes were selected for further examination (Fig. 7g).

For apoptosis analysis, HeLa cells were treated with either compound alone (500 nM AH057, 1 μM SGI-1027) or two compounds in combination for 24 h. Treated cells were stained with annexin V-PE and 7-AAD, and assayed for their apoptotic cell death by flow cytometry. An increase in the percentage of apoptotic cells was observed in HeLa cells treated with either AH057 or SGI-1027 alone, and the combined treatment further increased the percentage of apoptotic cells (Fig. 7a). Quantitative data clearly showed that the combined treatment resulted in a higher percentage of apoptotic cell death, with the early apoptosis percentage increased in a synergistic manner (Fig. 7b). Next, we investigated the underlying apoptosis signal pathways using WB analysis. When the cleavage of poly (ADP-ribose) polymerase (PARP) and Caspase-3 was monitored, moderately and dramatically increased cleavage products were detected in AH057-treated cells and drug combination-treated cells, respectively (Fig. 7c). In addition, the expression of antiapoptotic factors Bcl-XL and survivin was examined by WB. Even though the decrease of Bcl-XL and survivin was readily detectable in HeLa cells treated with AH057 alone, the addition of SGI-1027 to AH057 further enhanced the decrease of Bcl-XL and survivin (Fig. 7c). Collectively, these data suggested that the apoptotic cell death induced by combination treatment of AH057 and SGI-1027 was associated with Caspase-3 mediated-PARP cleavage and the reduction of Bcl-XL and survivin.

As stated above, we found that AH057 can induce G1/S arrest of CC cells (Fig. S2). To find out if SGI-1027 can potentiate this cell-cycle arrest effect, we examined the combined effect of AH057 and SGI-1027 on cell-cycle progression of HeLa cells. Cells were treated with either drug alone (500 nM AH057, 1 μM SGI-1027) or in combination for 24 h, fixed in 70% of ethanol, and stained with PI. DNA content was measured by flow cytometry (Fig. 7d). SGI-1027 provoked an accumulation of HeLa cells in the S phase, while combination treatment induced a higher percentage of cells in G1 phase than either single-drug treatment, implicating a G1/S arrest (Fig. 7e). The expression levels of several key cell-cycle regulators such as c-Myc, Cyclin D1, Cyclin A2, and Cyclin B1 were determined by WB. C-Myc was not significantly changed in cells treated with AH057 singly or in combination. In contrast, the decrease in Cyclin D1, Cyclin A2, and Cyclin B1 protein levels was discernable with AH057 treatment alone, and the combination treatment further potentiated the decrease in these cyclin protein levels (Fig. 7f) that may be responsible for the cell-cycle arrest. Furthermore, qRT-PCR analysis showed that the transcriptional pattern of the selected genes in drug combination group largely followed that in the AH057 group, and combination treatment exhibited synergistic downregulation of some genes (e.g., BAX, BCL-XL, BCL-2, MDM2, Cyclin A2, Cyclin D3, and CDK2) where SGI-1027 treatment alone showed no changes or even slight upregulation (Fig. 7g).

## Discussion

CC is a malignant tumor that severely harms the health of women. Its stages are defined by the International Federation of Gynecology and Obstetrics^28^. Up to date, the long-term prognosis of CC is still very poor, with an expected 5-year survival rate of <10%. Developing novel chemotherapies and targeted therapies holds the promise of improving the prognosis of CC patients^29^. Recently, emerging studies^30–32^ indicated the potential of JAK/STAT pathway as an ideal prognostic and therapeutic target for CC.

JAK/STAT signaling pathway is an essential pathway for signal transduction of various growth factors and cytokines. Constitutive activation of the JAK1/STAT3 pathway is frequently observed in cancer cells^33,34^ while JAK2/STAT3 is closely associated with cytokine expression, cell apoptosis, and proliferation^35,36^. Despite the well-established roles of the JAK/STAT signaling in cancer cell survival, its therapeutic applications in CC remains largely unexplored. In this study, by virtual screening, we identified and validated a novel JAK1/2 inhibitor AH057 that exhibited most potent antitumor efficacy against CC cells over other cancer cells. Importantly, AH057 did not affect the protein levels of NF-κB p65 and pSer365-p65, one of the downstream targets of STAT3 in esophageal squamous cell carcinoma cells^37^. Nor did AH057 affect the protein levels of AKT or pSer473-AKT, the critical signaling node indirectly regulated by JAK2^27^. These results, together with other accompanying results, suggested that in the cellular context of CC, AH057 specifically blocked JAK1/2 and its downstream signaling pathways. It remains unclear, however, why AH057 works most efficaciously on CC cells and particularly on HeLa cells, and further studies are warranted to reveal the underlying mechanisms.

Previous studies have shown that inhibiting the JAK/STAT pathway alone was not sufficient to completely suppress the propagation of CC cells in vitro^38^. This could be due to compensations by alternative growth and survival pathways activated via autocrine/paracrine signals. In this regard, combination therapy may be an effective strategy to tackle the problem. For instance, it has previously been reported that targeting IL-6/JAK2/STAT3 signaling axis with FDA-approved agents, alone or combinatorially with HER2 inhibitors substantially repressed the tumorigenic properties of HR−/HER2+ breast cancer (BC)^39^. Besides, although inhibiting the SRC pathway or STAT3 had negligible effects on each other, combined treatment with both JAKi and SRCi led to substantial inhibition of both pathways and exhibited much stronger antitumor activities against human ovarian cancer cells^40^. To search for compounds that may act synergistically with AH057, we screened a chemical library comprising 2094 small-molecule compounds, and identified the DNMT1 inhibitor SGI-1027 as the best hit.

The DNMT family that includes DNMT1, DNMT3A, and DNMT3B, is mainly involved in mediating methylation of CpG islands^41^. DNMT1 cooperates with critical genes for radio- and chemoresistance in cancer cells, to regulate promoter methylation of tumor suppressor genes (TSGs). Inhibiting DNMTs has been found to attenuate tumor cell growth and induce cell apoptosis. Therefore, DNMT inhibitors (DNMTi) are now considered as potential anticancer agents for cancer therapy^42^. SGI-1027, a non-nucleoside DNMTi, belongs to a novel class of relatively stable, highly lipophilic quinoline-based (monoquaternary pyridinium analogue) small-molecule inhibitors of DNMT, mainly DNMT1^43^. SGI-1027 may inhibit DNMT activity, induce the degradation of DNMT1 and thereby reactivating TSGs^44^. Besides, the DNMT1 inhibitor 5-aza-2′-deoxycytidine (5-Aza-2-dc) alleviated Ochratoxin A (OTA)-induced cytotoxicity and DNA damage in PK15 cells^45^, and another DNMT1 inhibitor 5-Azacytidine prevented cisplatin-induced nephrotoxicity in male SD rats^46^, indicating that the relatively low cytotoxicity of DNMT1 inhibitors may position them as ideal partner compounds for combination therapies.

Here, we discovered that AH057 and SGI-1027 synergistically induced CC cell apoptosis by simultaneously enhancing the cleavage of PARP and Caspase-3 and decreasing the expression of Bcl-XL and Survivin. A previous report showed that the DNMTi Zebularine increased the apoptosis rate and the proportion of medulloblastoma cells in the S phase^47^. The repositioned JAK/STAT inhibitor Zelnorm caused G1 cell-cycle arrest of a variety of cancer cells^48^. These results prompted us to assess the cell-cycle state of the AH057/SGI-1027-treated cells. Remarkably, we found that AH057 treatment alone significantly reduced the expression of Cyclin D1, B1 and A2, which blocks G1/S entry, G2/M transition and both, respectively. Compared with the flow cytometry data, more severely repressed Cyclin D1 and A2 may be responsible for the G1 arrest. In contrast, SGI-1027 treatment alone slightly reduced the expression of Cyclin D1, B1, and A2, but exhibited S phase arrest. Since S phase cells contain high levels of methylated DNMT1 proteins^49^ and DNMT1 can mediate transcriptional repression by forming a complex at replication foci in S phase^50^, SGI-1027 treatment may disrupt normal functionality of DNMT1 that is required for S-phase exit. As expected, the combination of AH057 and SGI-1027 mainly induced prolonged G1 phase and shortened G2 phase.

The superior synergistic effects of AH057 and SGI-1027 beg the question about the underlying molecular mechanisms. Some reports indicate that JAK/STAT and DNMTs may form a reciprocal positive regulatory loop. For instance, inhibiting DNMT1 and DNMT3A by 5-Aza-2dc attenuated the phosphorylation of JAK2 and STAT3^51^, possibly by upregulating the expression of SOCS3, the known suppressor of JAK2 and STAT3 phosphorylation^45^. On the other hand, the binding of STAT3 to the promoter of DNMT1 augmented DNMT1 transcription in malignant T-lymphocytes^52,53^. In carcinoma-associated fibroblasts, DNMT3B methylated CpG sites of the promoter of SHP-1 phosphatase and abrogated SHP-1 expression, leading to constitutive phosphorylation of JAK1. Sustained JAK1/STAT3 signaling was maintained by DNMT1-dependent downregulation of the SHP-1^54^. Moreover, in chronic myeloid leukemia blast cells, the long noncoding RNA MEG3 interacted with multiple proteins in a large complex comprising DNMT1, JAK2, TYK2, STAT3, and HDAC1^55^. More recently, IL-6/JAK1-mediated STAT3 phosphorylation was found to promote the transcription of DNMT3B and OCT4 in hepatocellular carcinoma cells, which can further up-regulate the transcription of DNMT1^56^. Collectively, these studies indicated that JAK/STAT pathway may work collaboratively with DNMTs in maintaining the survival and proliferation of cancer cells, providing a sound rationale for combining a JAKi with a DNMTi. Interestingly, chloroquine, an autophagy inhibitor, was found to significantly lower the proportion of cancer stem cells in triple negative BC, by concurrently reducing the expression of JAK2 and DNMT1^36^. Of note, although cervical squamous cell carcinoma (CESC) patients with higher DNMT1 mRNA levels had shorter overall survival (OS) (Fig. S9A), there was no significant correlation between JAK1 or JAK2 mRNA levels and OS (Fig. S9B, C), indicating that mRNA levels may be discordant with protein levels and enzymatic activities for JAKs. Thus, when the synergistic effects of JAKi and DNMTi on target gene expression are to be assessed, both mRNA levels and protein levels of the target genes need to be examined.

In summary, we identified a novel JAK1/2 inhibitor AH057 that potently suppresses the propagation of CC cells. Simultaneously inhibiting the JAK/STAT and DNMT1 pathways by combined treatment with AH057 and SGI-1027 exhibited strong synergism in blocking the proliferation of CC cells and inducing their cell-cycle arrest and apoptotic death. The reciprocal positive regulation between the JAK/STAT and DNMT1 pathways is likely to account for such synergism, and both pathways converge on multiple downstream effectors such as Bcl-XL/Survivin, PARP/Caspase-3, and Cyclin A/Cyclin D that are dually targeted by the drug combination (Fig. 7h). Therefore, combination therapies to concurrently block multiple interacting signaling/survival pathways hold great promise in treating advanced CC and other malignant human cancers.

## Materials and methods

### Virtual screening strategy

#### Protein preparation

The X-ray crystal structure of the JAK2 kinase domain (PDB code: 3RVG) was employed as the template for virtual screening. All water molecules were removed, and the missing hydrogen atoms were added by Accelrys Discovery Studio (version 2.5, Accelrys, San Diego, CA, USA) using the “Prepare Protein” tool. The activator pocket was defined by the crystal ligand using the “Find Sites as Volume of Selected Ligand” tool.

#### Ligand structures

The SPECS database (http://www.specs.net), which contains ~350,000 compounds, was used for the screening. The 20,000 ligands were prepared using the “Prepare Ligand” module by the CHARMM force field^25^.

#### Virtual screening procedure

First, LibDock mode was used as the screening engine. After docking, top-ranking molecules were selected based on their LibScore. The best-scored molecule compounds were overlaid with the crystal structure ligand for visual inspection. Finally, nine compounds were selected and purchased from SPECS^57^.

### Cell lines and reagents

All cell lines were purchased from Chinese Academy of Sciences and cultured in Roswell Park Memorial Institute 1640, Eagle’s Minimum Essential Medium (MEM) or Dulbecco’s modified Eagle’s medium (DMEM) (Gibco/ThermoFisher Scientific, Waltham, MA, USA) supplemented with 10% fetal bovine serum (FBS, Gibco) and 1× penicillin and streptomycin (Gibco) at 37 °C in a 5% CO2 incubator. Cells were free of mycoplasma contamination based on routine tests every 3 months.

### Establishment of stable cell pool

Sequences encoding human JAK1-JH1 domain, JAK2-JH1 domain, and TYK2-JH1 domain were cloned into pLKO-puro lentiviral expression vector separately. Each of the above constructs was transfected into HEK293T cells combined with pMD2.G and psPAX2 helper vectors for virus packaging. After 48 h packaging, mature viral particles were collected and applied to HEK293T cells, overnight-cultured cells were replaced with fresh media and cultured for another 24 h. Stable cell pools were developed by puromycin (2 mg/ml) selection for 7 days.

### Cell viability assay

Exponentially-growing cells were treated with AH057 (dose range = 0.0005–100 μM) and/or other compounds for 3 days. Cell viability was determined by Cell Counting Kit-8 (Beyotime #C0039) assay according to the manufacturer’s instruction. The half maximal inhibitory concentrations (IC50s) of the compounds were determined using Prism software (GraphPad).

### Cell invasion assay

The CC cell lines were chosen for the cell invasion assay due to their high metastatic potential. Transwell chambers were suspended in serum-free medium (Corning Inc., Corning, NY, USA) and 100 μl of cells (1–2 × 10^5^ per well) were added to the upper chambers of the 24-well plates. Concurrently, AH057 was diluted with 0.6 ml of 20% FBS–DMEM complete medium (final drug concentrations for CC cells were indicated) was added to the lower chambers. After 48 h, the invaded cells in the lower chambers were fixed, stained with 0.1% crystal violet solution and then photographed (magnification, ×100).

### Western blot and qRT-PCR analysis

The protein samples from stimulated or transfected cells were extracted by using the Laemmli Sample Buffer (Bio-Rad Laboratories, Inc., USA). The concentrations of protein samples were quantified by using the BCA™ Protein Assay Kit (Beyotime #P0012). Antibodies purchased from Cell Signaling Technology (Beverly, MA) included: phospho-STAT3 (Tyr705) (#9145), phospho-STAT3 (Ser727) (#49081), STAT3 (#4904), phospho-JAK2 (Tyr1007/1008) (#3771), JAK2 (#3230), phospho-JAK1 (Tyr1034/1035) (#74129), JAK1 (#3344), phospho-TYK2 (Tyr1054/1055) (#68790), TYK2 (#14193), phospho-STAT1 (Tyr701) (#7649), STAT1 (#14994), phospho-AKT (Ser473) (#4060), AKT(Pan) (#4821), phospho-NF-κB p65 (Ser536) (#3033), NF-κB p65 (#8242), cyclin D1 (#2978), cyclin A2 (#4656), cyclin B1 (#12231), Caspase-3 (#29629), Bcl-xL (#2762), and Survivin (#2803). PARP antibody and GAPDH antibody were purchased from Beyotime (#AP102) and Genscript (#A00192), respectively. WB analysis was conducted as previously described^58^. Three independent experiments were performed using samples collected at different days. qRT-PCR was conducted as described previously^59^. All the primers used were listed in Fig. S1b.

### Cell-cycle analysis

Compound-treated cells were collected, fixed in 70% prechilled ethanol at 4 °C overnight, stained with propidium iodide (PI), and examined by FACS (fluorescence-activated cell sorting) analysis (Becton Dickinson, Mountain View, CA).

### Apoptosis assay

Apoptosis was quantified using the Annexin V-PE and 7-AAD Apoptosis Detection Kit I (BD Pharmingen, La Jolla, CA, USA) according to the manufacturer’s instructions. Briefly, 1 × 10^6^ of cells treated with vehicle, AH057 and SGI-1027 alone or in combination, were washed in ice-cold phosphate buffered saline (PBS), resuspended in 100 μl of binding buffer, and incubated with 5 μl of Annexin V-PE and 5 μl of 7-AAD for 15 min in a dark room following the manufacturer’s instruction. Flow cytometric analysis was immediately performed using a FACS Calibur flow cytometer (Becton Dickinson, Franklin Lakes, NJ, USA). Early apoptotic cells are Annexin V-PE positive and 7-AAD negative, while late apoptotic cells are positive for both dyes. Viable cells with intact membranes exclude 7-AAD, and are negative for both dyes.

### Colony formation assay

The cells were plated at a density of 250 cells per 60 mm dish and cultured in MEM medium overnight before being replenished with fresh medium supplemented with the indicated concentrations of AH057. After culture for 14 days, the cells were fixed with 4% paraformaldehyde (PFA) and stained with 1% methylene blue. The visible colonies were counted. All experiments were performed in triplicate, and the values are presented as the mean ± SD.

### Mouse xenograft tumor models

BALB/c nude mice (female, 3–4 weeks old) were purchased from Shanghai Experimental Animal Center, Chinese Academy of Science. The Experimental Animal Ethics Committee of Zhejiang University has approved the animal protocol. HeLa cells were injected subcutaneously at 1.5 × 10^6^ cells/mice in a 0.2 ml volume of PBS buffer, and after tumor formation, the mice were randomly divided into 2–4 groups with six mice per group. A random number table method was used to assign mice. Experimental treatments with AH057, SGI-1027 (50 mg/kg per day), or their combo were performed by daily administration of the compound in 200 μl volume of DMSO and corn oil (1:9, v/v) via oral gavage. Control mice were treated with the vehicle only. Tumor growth was measured every 3 days using vernier calipers, and the tumor volumes were calculated as a product of 1/2 × length × width × height. The mice were weighted and carefully monitored for the appearance of any side effects, and sacrificed at the end of the experiments for histological analysis of the lesions. No blinding was conducted throughout the experiments.

### Drug combination screening with a biologically annotated compound library

HeLa cells were seeded at 4000 cells/well in 96-well black plates. 1 μM of one of the 2094 small-molecule compounds purchased from the Selleck libraries (L2000-Z156796 and L2000-01, Selleck Chemical, USA) that have diverse functions, structures, and cellular targets, were added individually into each well together with either 500 nM AH057(combination condition) or with dimethylsulfoxide (DMSO) (single condition), and cells were incubated overnight. The combined drug effect was determined by Bliss independence analyses, which was calculated by the equation: C = (A + B) − (A × B) where A and B are the fractional growth inhibitions of drug A and B at a given dose. The difference between the Bliss expectation and the observed growth inhibition of the combination of drugs A and B at the same dose is the “Delta Bliss”. Delta Bliss scores were summed across the dose matrix to generate a Bliss sum. Bliss sum = 0 indicates that the combination treatment is additive (as expected for independent pathway effects), Bliss sum > 0 indicates activity greater than additive (synergy), and Bliss sum < 0 indicates the combination is less than additive (antagonism).

### TUNEL staining in vivo

The TUNEL assay was carried out to evaluate apoptosis using the in situ apoptosis detection kit (Takara Bio, Inc.). Briefly, The sections were incubated with 50 µl of a labeling reaction mixture (consisting of TdT enzyme 5 µl + Labeling Safe Buffer 45 µl) in a 37 °C humidified chamber for 90 min. Immunoreactivity was visualized by immersing the sections in DAB for 10 min at room temperature. Next, the sections were counterstained with hematoxylin for 10 s and dehydrated in 100% ethanol, which was replaced by clear Plus. EXCEL Mount (Falma) served for mounting. The sections were rinsed three times in PBS between all the steps. The proportions of apoptotic cells were evaluated by counting the TUNEL-positive cells among all tumor cells, avoiding necrotic tumor areas, in a minimum of seven visual fields in each individual section under a light microscope (with ×200 magnification).

### Immunohistochemical analysis

The immunohistochemical procedures were performed using a streptavidin-peroxidase (SP)-conjugated method according to the manufacturer’s instructions. Paraffin-embedded tissues were cut into 4 μm-thick serial sections; the tumor sections were sealed in 5% blocking serum at room temperature for 30 min and probed with primary antibodies overnight at 4 °C. Sections were stained with primary antibodies, including rabbit anti-Ki67 (1:100 dilution). At least three random fields from each section were examined.

### Statistical analyses

All quantitative results are expressed as mean values ± S.D. All statistical analyses were conducted using the GraphPad Prism 7.0 statistics software released by GraphPad Software, Inc. The statistical significance of normally distributed or non-normally distributed data was evaluated using the two-tailed unpaired Student’s test or Mann–Whitney test, respectively, and differences were considered significant at *P < 0.05; **P < 0.01; ***P < 0.001.

## Supplementary information

Supplementary figure legends

Supplementary figure 1

Supplementary figure 2

Supplementary figure 3

Supplementary figure 4

Supplementary figure 5

Supplementary figure 6

Supplementary figure 7

Supplementary figure 8

Supplementary figure 9

Supplementary figure 10
