# Supplementary material for: Combined inhibition of JAK1/2 and DNMT1 by newly identified small-molecule compounds synergistically suppresses the survival and proliferation of cervical cancer cells
Source: Cell Death Dis. 2020 Sep 7;11(9):724. doi: 10.1038/s41419-020-02934-8 (PMC7476923; doi:10.1038/s41419-020-02934-8)
Supplement: Supplementary file 1 — Supplementary figure legends [file 41419_2020_2934_MOESM1_ESM.docx]

**Supplementary figure legends**

**Figure S1.** (A) SPECS ID numbers for nine compounds shown in Fig. 1A. (B) The primer sequences used in this work. (C) ^1^H-NMR spectrum for AH057. (D) ^13^C-NMR spectrum for AH057. (E) HeLa, DU145 and HepG2 cells were serum-starved, pretreated with AH057 for 1 hour and stimulated by IFNα (250 ng/ml) for 4 hours. Relative mRNA levels of IRF2 were determined by qRT-PCR.

**Figure S2.** AH057 induced apoptotic death and cell cycle arrest of CC cells. (A) Flow cytometry analysis of apoptotic cell death in HeLa cells upon AH057 treatment. Cells were treated with 1 μM of AH057 for 24 h, stained with annexin-PE(x-axis)/7-AAD(y-axis), and analyzed by flow cytometry. (B) Cells that underwent early apoptotic cell death (bottom right quadrants) and late apoptotic cell death (top right quadrants) were calculated and plotted as percentages of total cells. (C) CC cells were treated with varying concentrations of AH057 for 24 h, fixed in 70% of ethanol, stained with propidium iodide, and subjected to flow cytometry for cell cycle analysis. (D) Quantitation of the percentages of cells in different cell cycle phases.

**Figure S3.** Effect of AH057 on HcerEpic, HeLa, CaSKi and SiHa cell migration determined by wound healing assay (n=3). Scale bars, 100 µm. Two-tailed unpaired Student’s t tests were used for statistical analyses. *P < 0.05, **P < 0.01 and ***P < 0.001 were vs. Control (the DMSO group).

**Figure S4.** AH057 and SGI-1027 combo potently induced cell death of HeLa cells. Adherent HeLa cells were cultured in 12-well plates, and treated with DMSO (Vehicle), 800 nM AH057, 500 nM SGI-1027, or 800 nM AH057+500 nM SGI-1027, for 3 days. Cells were collected and suspended with 100 μl PBS. Live and dead cells were counted by trypan blue exclusion using the Countess™ II FL Automated Cell Counter.

**Figure S5.** Combination effect of SGI-1027 and other JAK inhibitors against HeLa cells. Adherent HeLa cells were cultured in 12-well plates, and treated with DMSO (Vehicle), 1 μM SGI-1027 singly or in combination with varying concentrations of JAK inhibitors. Images were taken at day 3 after compound treatment.

**Figure S6.** Combination effect of AH057 and SGI-1027 on multiple cancer cell lines. Multiple cancer cell lines were cultured in 12-well plates, and treated with DMSO (Vehicle), 20 μM AH057, 1 μM SGI-1027 or 20 μM AH057+1 μM SGI-1027. Images were taken at day 3 after compound treatment.

**Figure S7.** Combination effect of AH057 and SGI-1027 on multiple cancer cell lines is dose-dependent. Multiple cancer cell lines were cultured in 12-well plates, and treated with DMSO (Vehicle), 1 μM SGI-1027 singly or in combination with varying concentrations of AH057. Images were taken at day 3 after compound treatment.

**Figure S8.** Combination effect of varying dose of AH057 and fixed dose of SGI-1027 on multiple cancer cell lines. Multiple cancer cell lines were cultured in 12-well plates, and treated with DMSO (Vehicle), a fixed dose of SGI-1027 (0.8 or 1 μM) singly or in combination with varying concentrations of AH057. The viability of cells treated with the compounds for 3 days was measured by CCK-8 assay.

**Figure S9.** Kaplan–Meier survival curve analysis with log-rank comparison was performed based on the mRNA levels of DNMT1 (A) , JAK1(B) and JAK2 (C) retrieved from the TCGA datasets.

**Figure. S10** Orally-administered AH057 and SGI-1027 synergistically inhibited propagation of HeLa cell xenografts *in vivo*. (A) The BALB/c nude mice were orally administered with indicated compounds as detailed in the Materials and Methods. At the end of the experiments, the executed mice bearing the tumors (left) and the excised tumors (right) were photographed and presented. (B) Effect of AH057, SGI-1027 and drug combo on body weight changes in xenograft mice. (C) Representative H&E staining images of heart samples from mice administered with AH057, SGI-1027 and drug combo. Scale bars, 100 µm. (D) Representative H&E staining images of kidney samples from mice administered with AH057, SGI-1027 and drug combo. Scale bars, 50 µm. Results shown in B were representative of 6 independent experiments. Two-tailed unpaired Student’s t tests were used for statistical analyses. *P < 0.05, **P < 0.01 and ***P < 0.001 were vs. Control (the DMSO group).
