# Supplementary figures and images for: Combined inhibition of JAK1/2 and DNMT1 by newly identified small-molecule compounds synergistically suppresses the survival and proliferation of cervical cancer cells
Source: Cell Death Dis. 2020 Sep 7;11(9):724. doi: 10.1038/s41419-020-02934-8 (PMC7476923; doi:10.1038/s41419-020-02934-8)

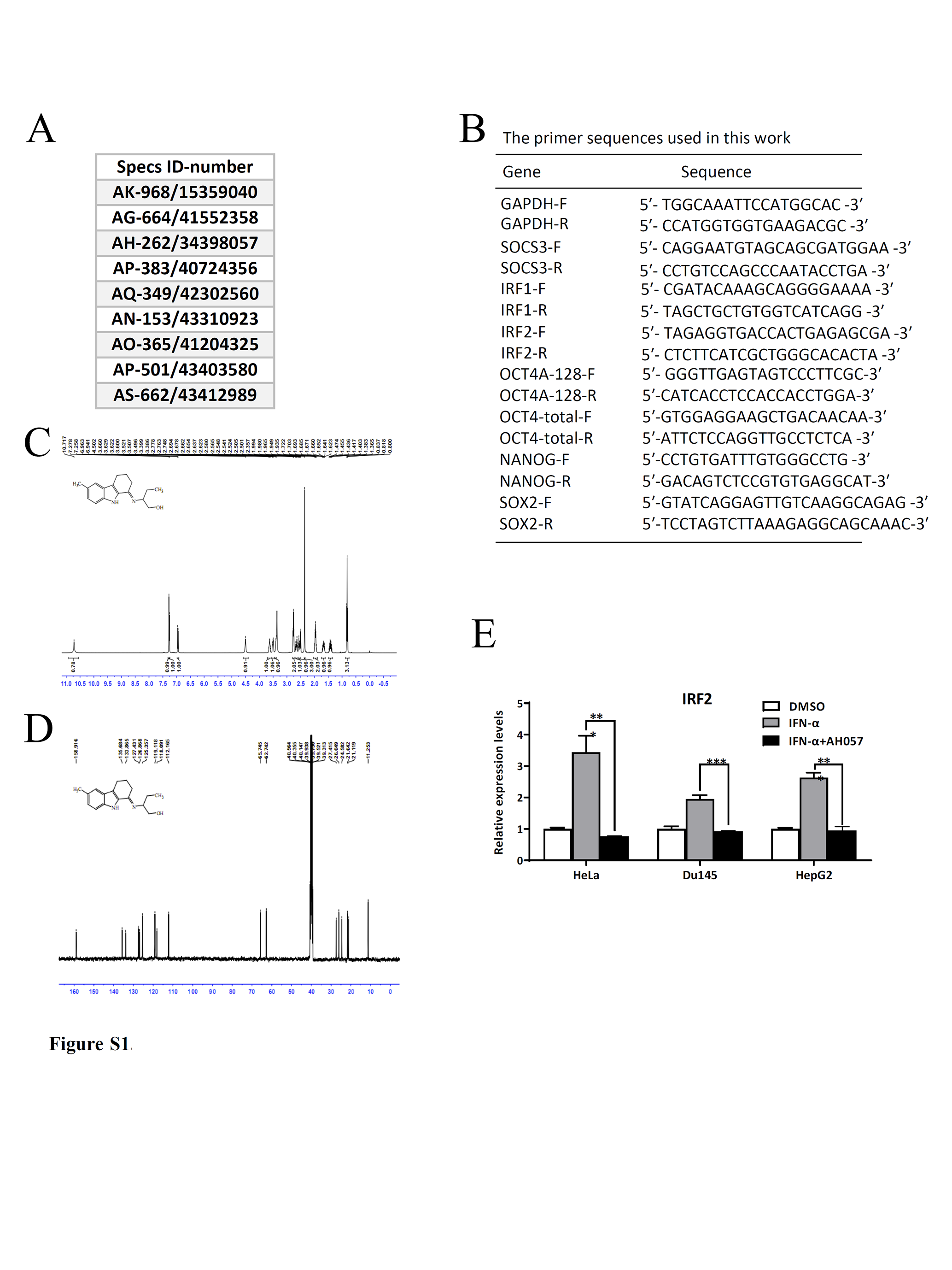

Supplement: Supplementary file 2 — Supplementary figure 1 [file 41419_2020_2934_MOESM2_ESM.png]

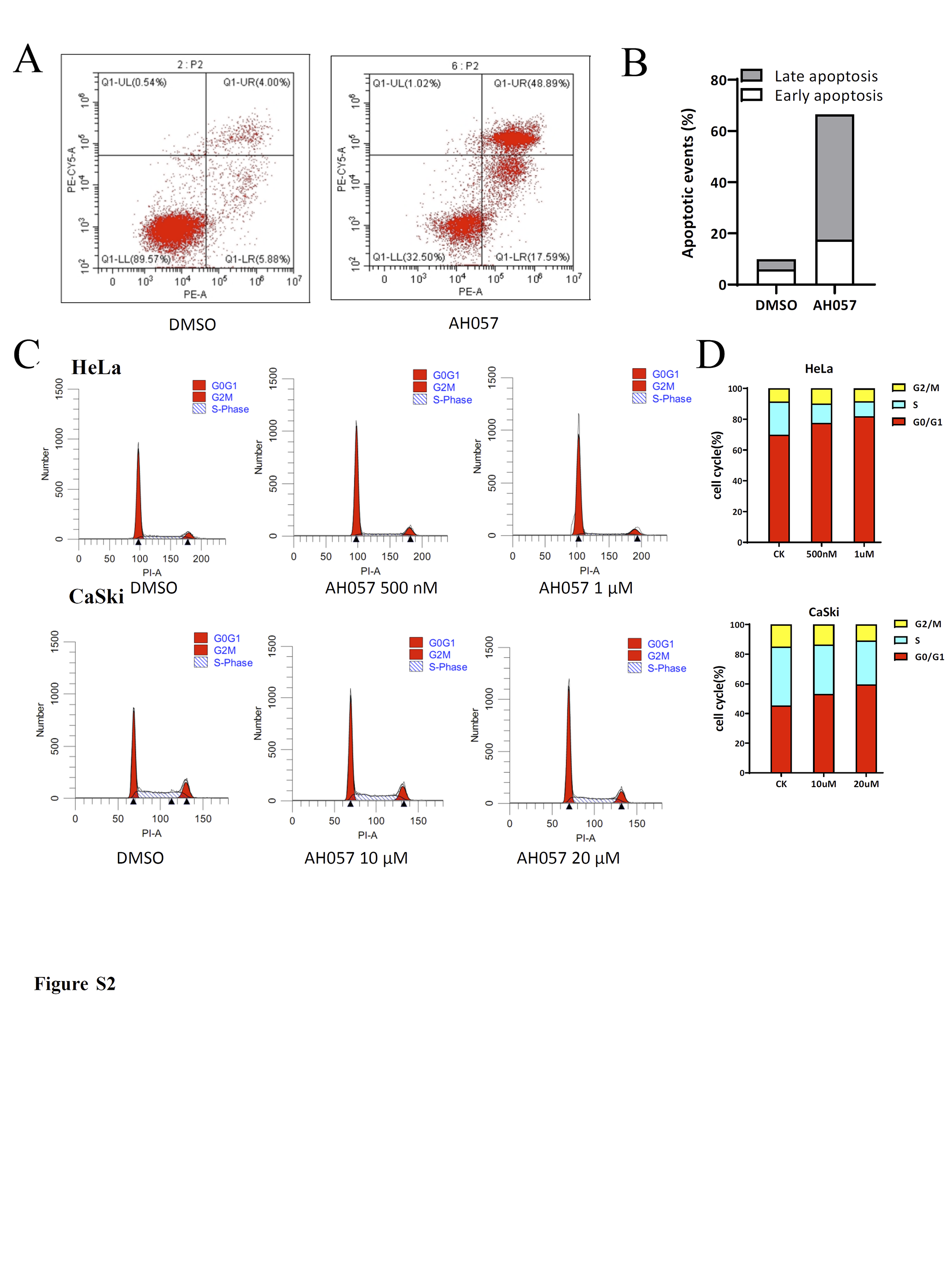

Supplement: Supplementary file 3 — Supplementary figure 2 [file 41419_2020_2934_MOESM3_ESM.png]

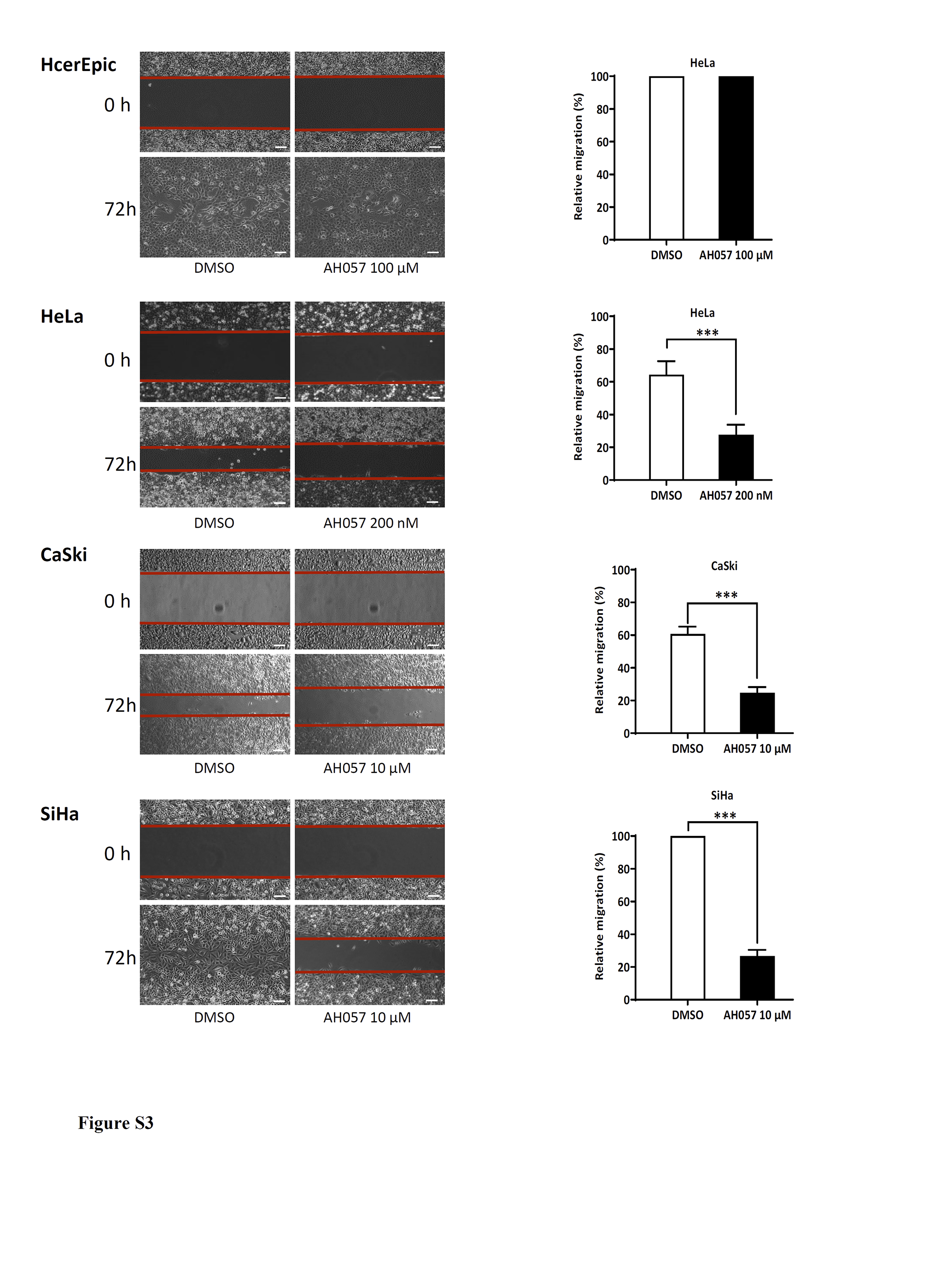

Supplement: Supplementary file 4 — Supplementary figure 3 [file 41419_2020_2934_MOESM4_ESM.png]

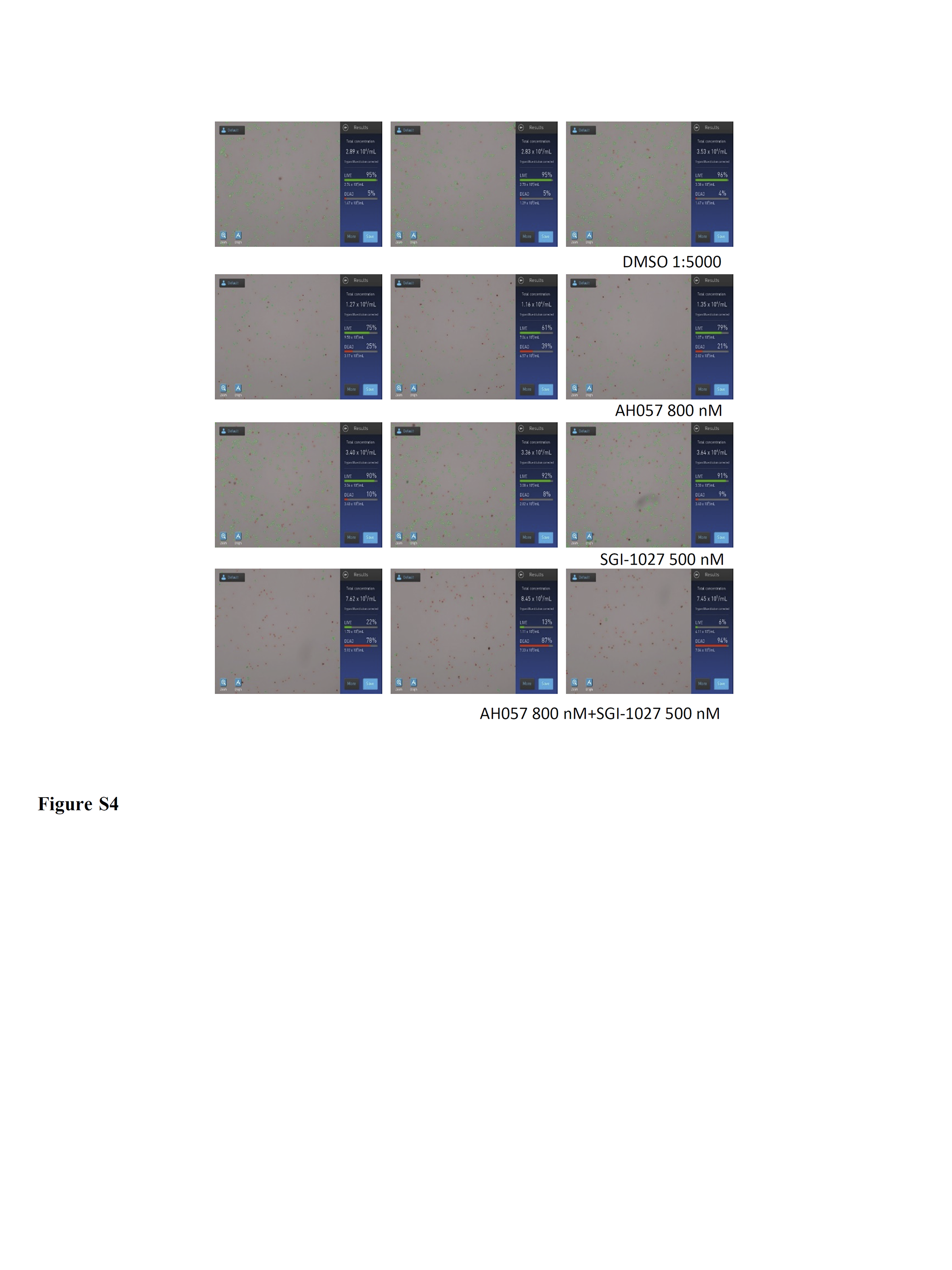

Supplement: Supplementary file 5 — Supplementary figure 4 [file 41419_2020_2934_MOESM5_ESM.png]

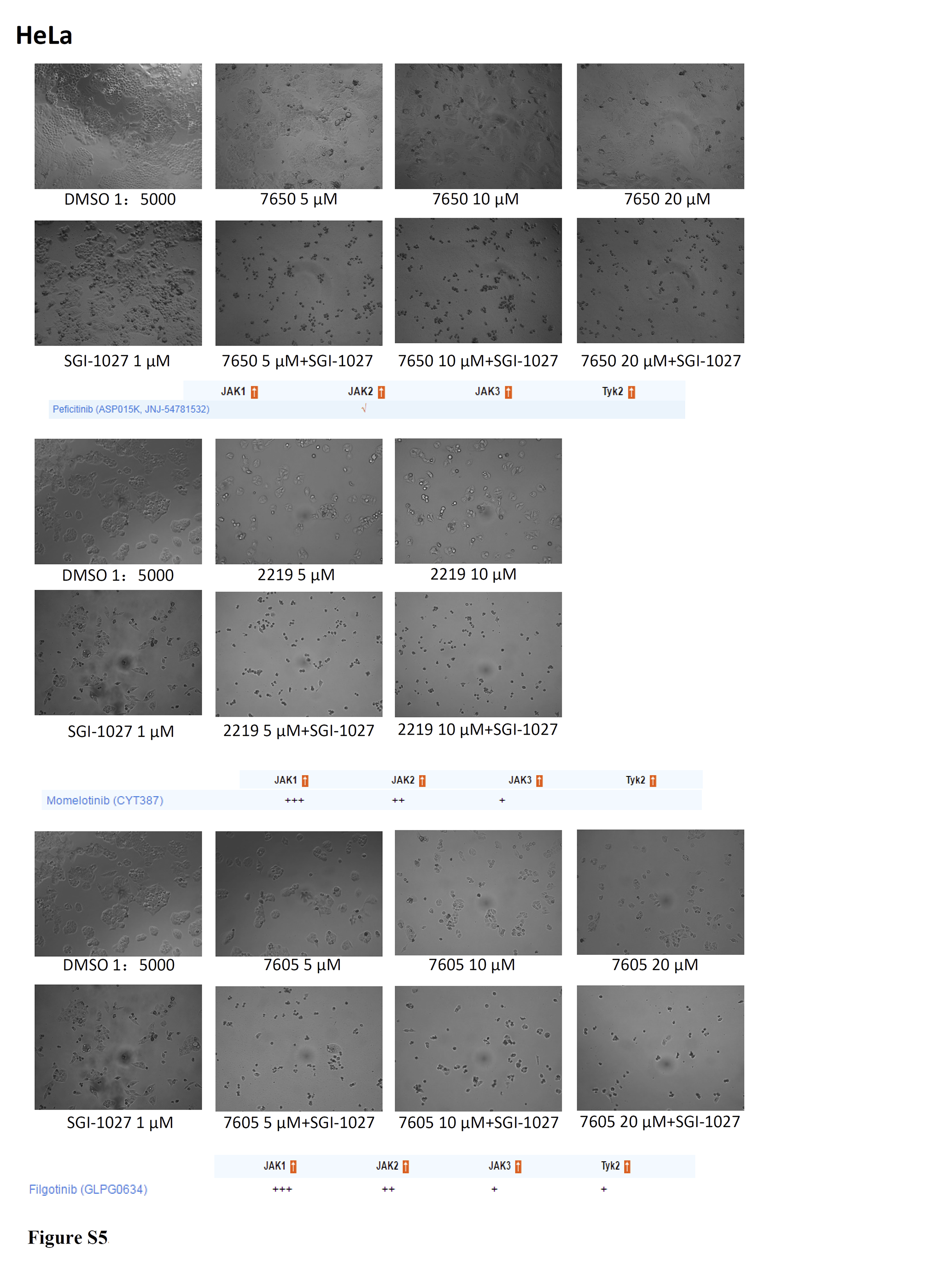

Supplement: Supplementary file 6 — Supplementary figure 5 [file 41419_2020_2934_MOESM6_ESM.png]

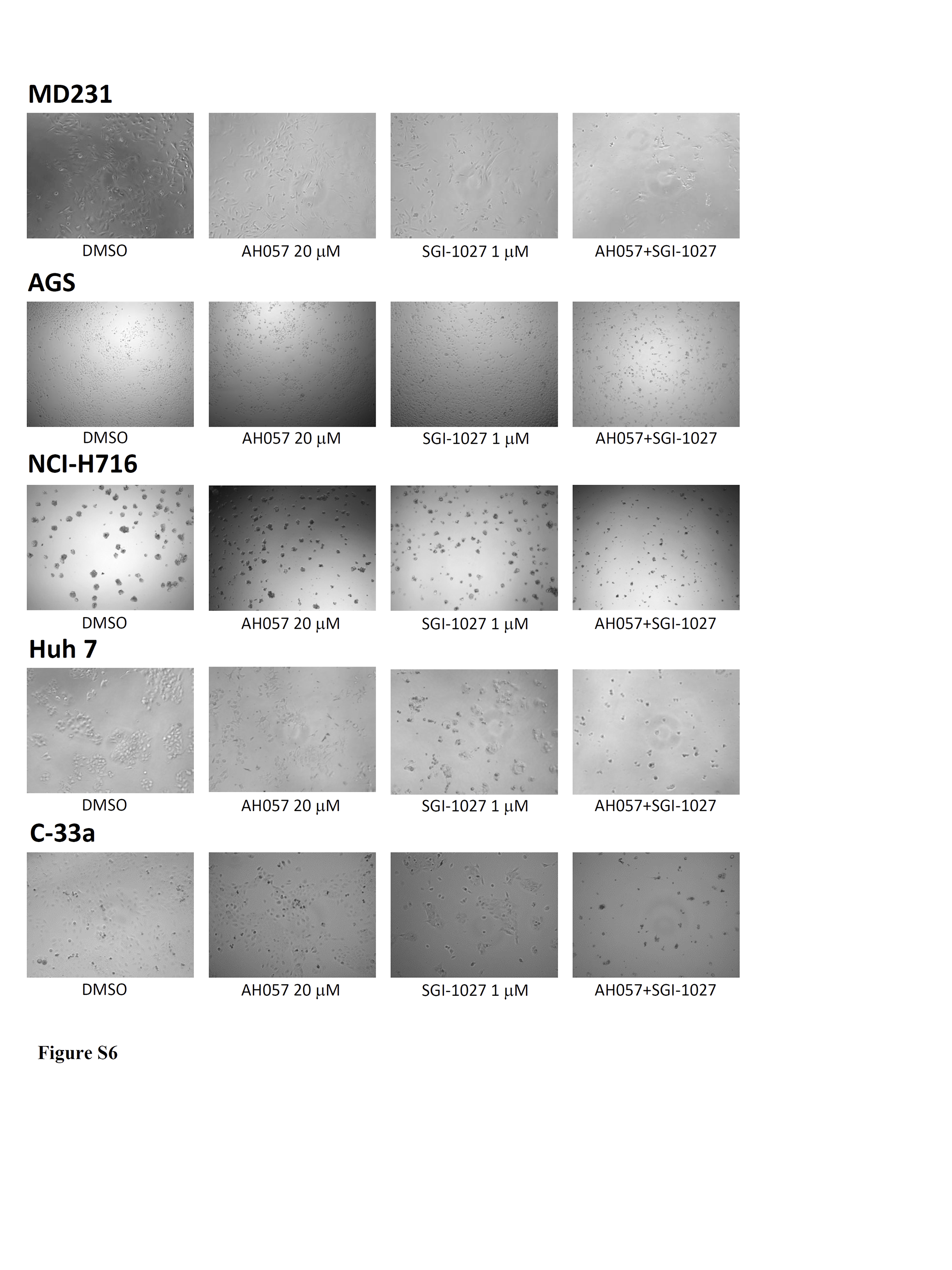

Supplement: Supplementary file 7 — Supplementary figure 6 [file 41419_2020_2934_MOESM7_ESM.png]

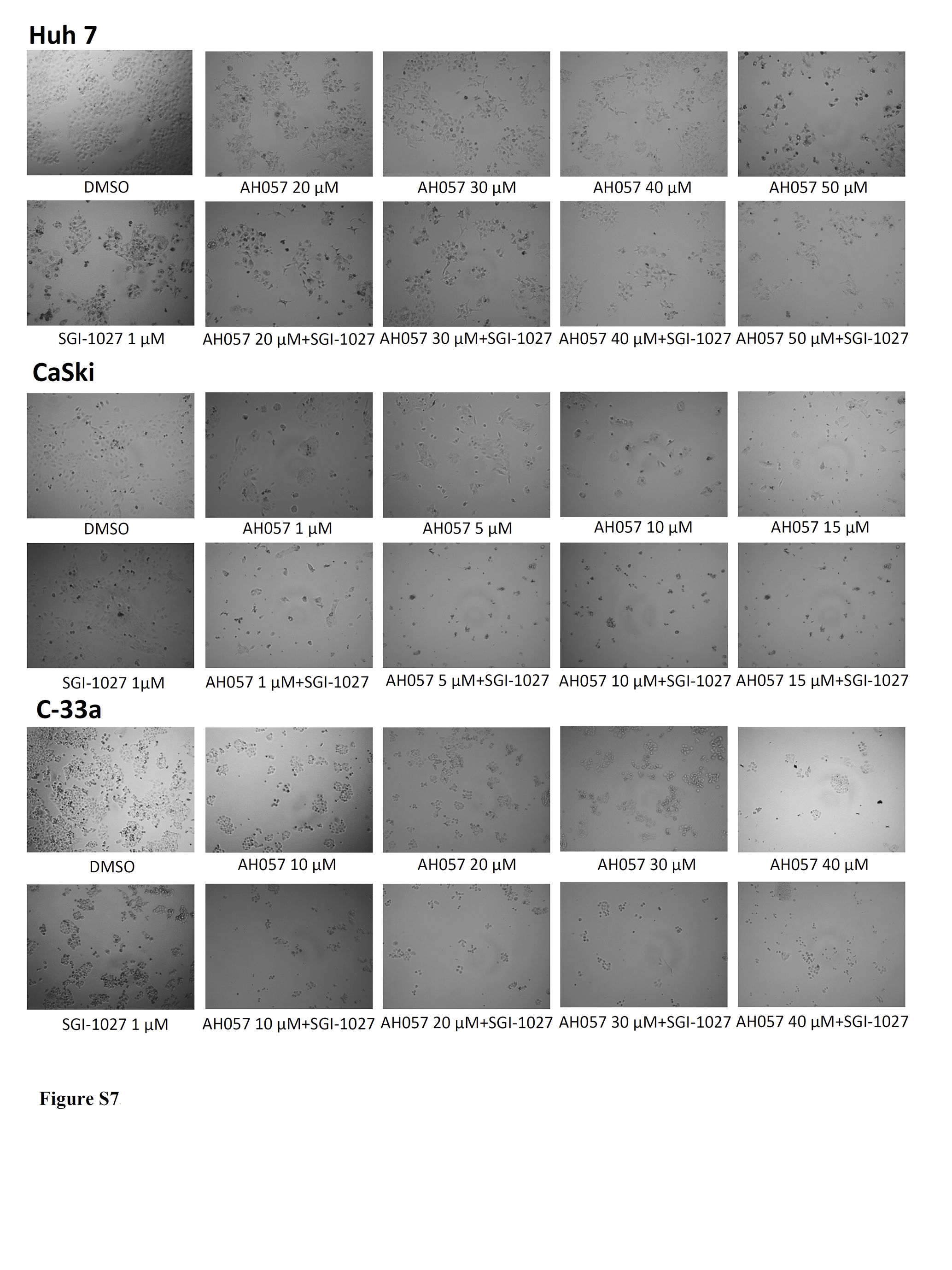

Supplement: Supplementary file 8 — Supplementary figure 7 [file 41419_2020_2934_MOESM8_ESM.png]

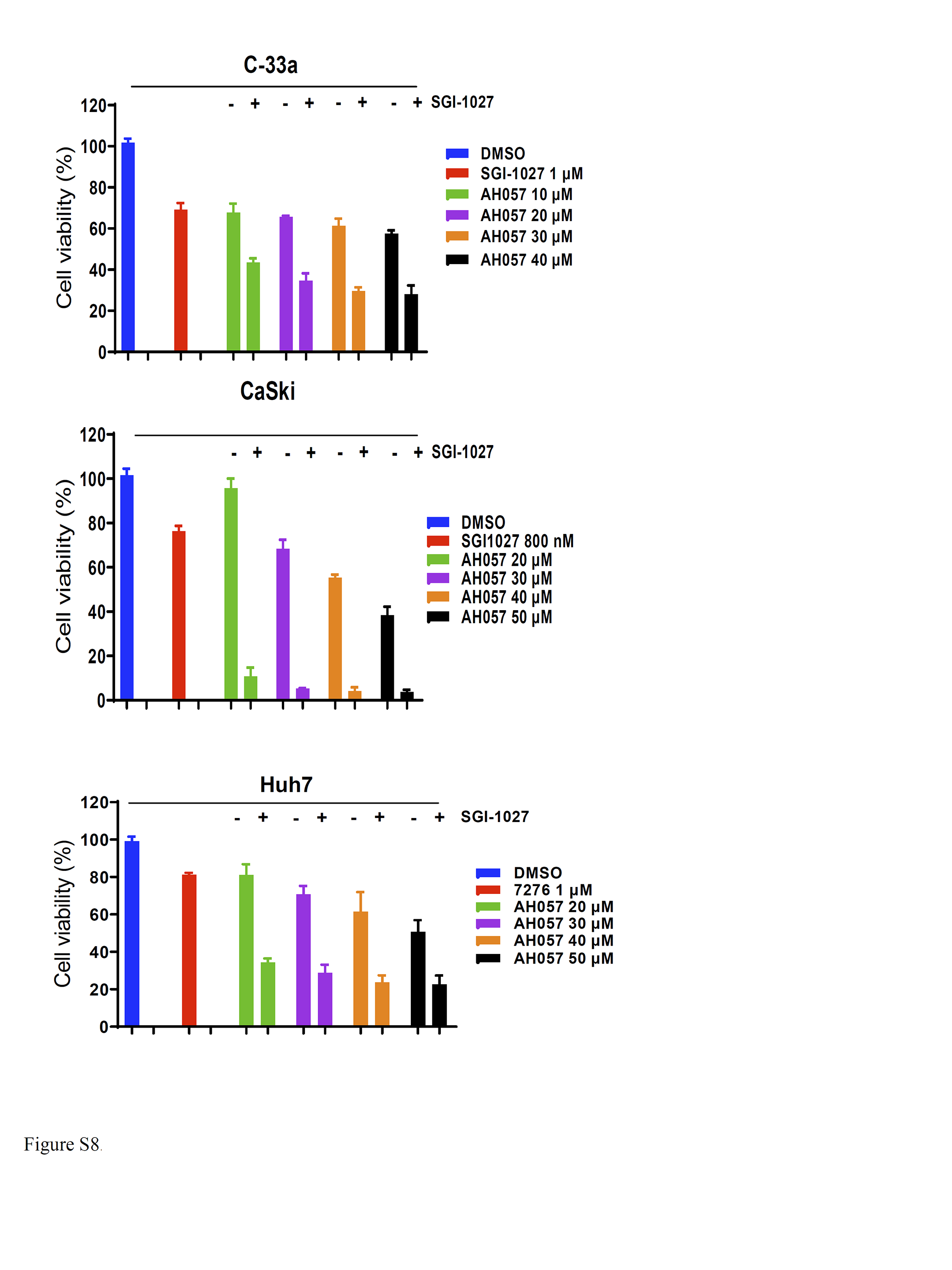

Supplement: Supplementary file 9 — Supplementary figure 8 [file 41419_2020_2934_MOESM9_ESM.png]

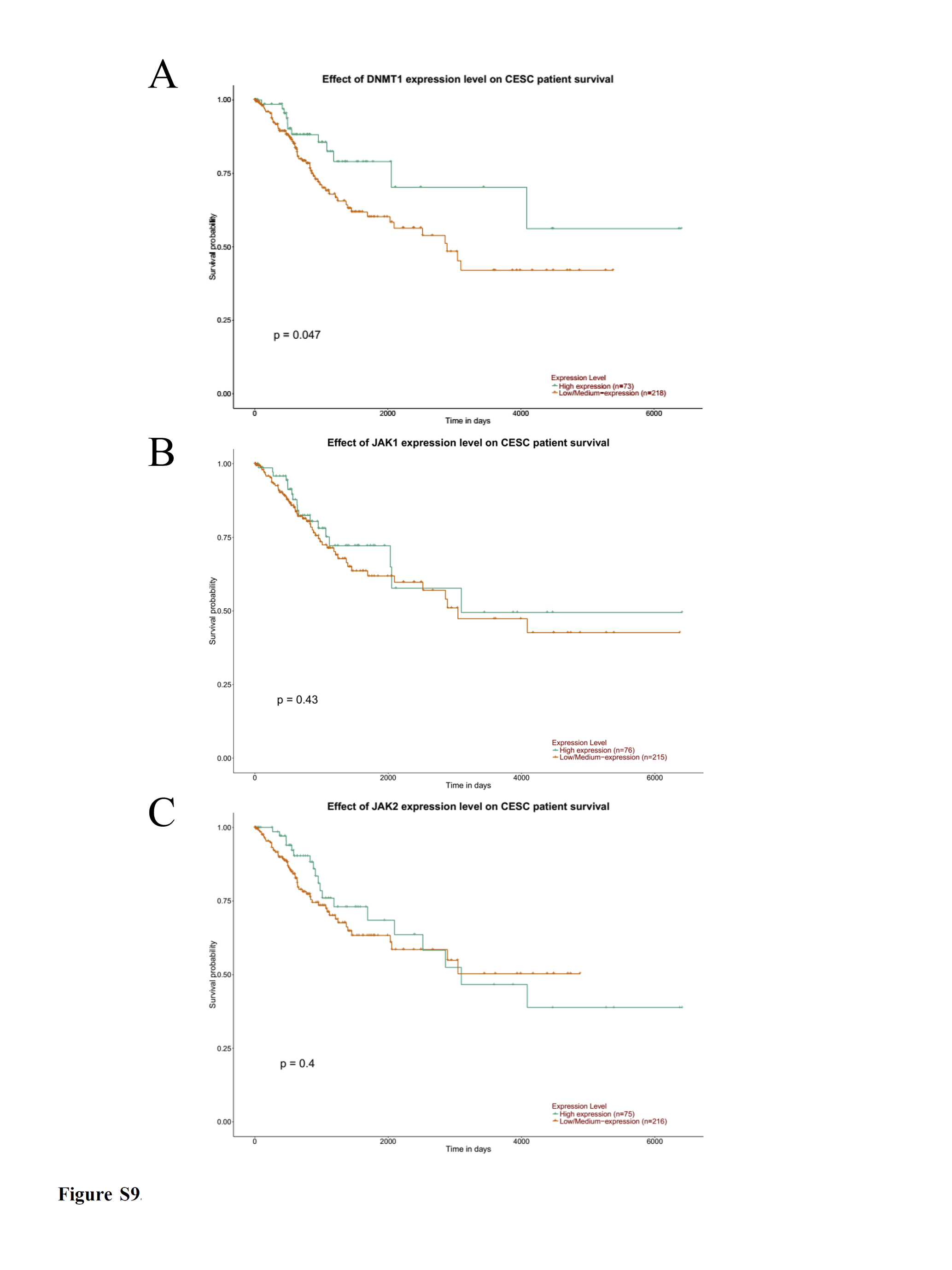

Supplement: Supplementary file 10 — Supplementary figure 9 [file 41419_2020_2934_MOESM10_ESM.png]

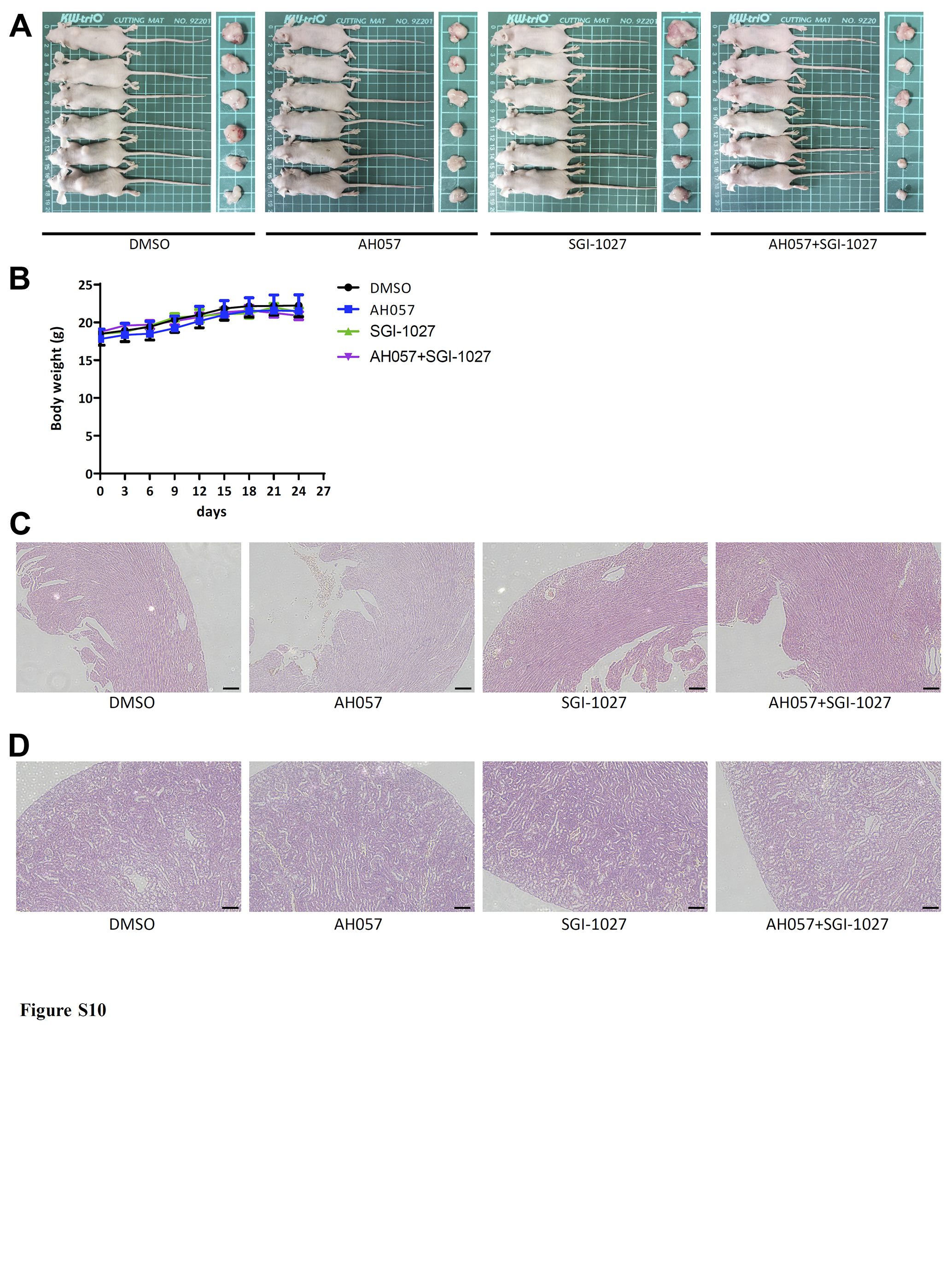

Supplement: Supplementary file 11 — Supplementary figure 10 [file 41419_2020_2934_MOESM11_ESM.png]
